# NLCD: A method to discover nonlinear causal relations among genes

**DOI:** 10.1371/journal.pcbi.1014570

**Published:** 2026-07-31

**Authors:** Aravind Easwar, Manikandan Narayanan

**Affiliations:** 1 Department of Computer Science and Engineering, Indian Institute of Technology (IIT) Madras, Chennai, India; 2 Centre for Integrative Biology and Systems medicinE (IBSE), Wadhwani School of Data Science and AI, IIT Madras, Chennai, India; 3 Sudha Gopalakrishnan Brain Centre, IIT Madras, Chennai, India; Michigan State University, UNITED STATES OF AMERICA

## Abstract

Distinguishing correlation from causation is a fundamental challenge in many scientific fields, including biology, especially when interventions like randomized controlled trials are infeasible and only observational data are available. Methods based on statistical tests of conditional independence within the Mendelian Randomization framework can detect causality between two observed variables that are each associated with a third instrumental variable. However, these methods for detecting causal relationships between traits (e.g., two gene expression or clinical traits associated with a genetic variant, all observed in the same population) often assume a linear relationship, thereby hindering the discovery of causal gene networks from genomics data. We have developed NLCD, a method for NonLinear Causal Discovery from genomics data based on nonlinear regression modeling and conditional feature importance scoring. NLCD uses these techniques to extend the statistical tests in an existing linear causal discovery method called the Causal Inference Test (CIT). We benchmarked NLCD against current state-of-the-art methods: CIT, Findr, and MRPC. On simulated datasets, NLCD performs comparably to most methods in detecting linear relations (Average AUPRC (Area Under the Precision-Recall Curve) of NLCD = 0.94, CIT = 0.94, Findr = 0.94, and MRPC = 0.99), and outperforms them in detecting nonlinear (sine and sawtooth type) relations between two genes (Average AUPRC of NLCD = 0.76, CIT = 0.60, Findr = 0.56, and MRPC = 0.73). When tested on a nonlinear subset of a yeast genomic dataset to recover known causal relations involving transcription factors, NLCD and CIT performed comparable to each other and slightly better than Findr and MRPC (Average AUPRC of NLCD = 0.82, CIT = 0.81, Findr = 0.71, and MRPC = 0.54). On application to a human genomic dataset, NLCD revealed active causal gene pairs (*IRF1* → *PSME1* and *HLA-C* → *HLA-T*) in the muscle tissue, and clarified the promises and challenges in discovering causal gene networks in tissues under *in vivo* human settings.

## Author summary

A central goal in biology is to discover causal mechanisms. But establishing causality between two biological factors (such as two genes) is difficult when interventions are expensive, impractical, or impossible as in human populations. Researchers therefore rely on observational genetic and other genomic data of hundreds of individuals to infer causal relationships between genes or other factors. Existing computational methods addressing this problem often assume that biological effects follow simple linear patterns. In reality, biological systems can exhibit more complex behaviors, which can cause important causal relationships to be overlooked. To address this limitation, we have developed a method that can detect causal relationships even when they are nonlinear. Our approach builds on existing causal discovery frameworks while using machine learning techniques to capture more complex patterns in the data. In extensive tests on simulated datasets, our method performs as well as current approaches for linear relationships and substantially better when relationships are nonlinear. When applied on yeast and human genomic datasets, our method recovered known causal interactions and identified biologically relevant gene pairs. The causal relations predicted by our method can serve as hypotheses to guide future experimental studies.

## 1. Introduction

Discovering causal relationships among a set of variables that constitute a complex system has been an active area of research in many fields, including bioinformatics, due to its many applications. For instance, distinguishing genes that cause a disease from those that are merely affected by the disease is important for finding targets for new drugs/therapies vs. diagnostic biomarkers for the disease. But an observed correlation between two traits such as gene expression traits does not imply causation, as it could also result from a confounder variable affecting both traits [1].

A gold standard solution for testing causality in the presence of confounding is a Randomized Controlled Trial (RCT), but RCTs are not always feasible and present additional challenges in an *in vivo* human setting [2]. An alternative solution for bivariate causal discovery is offered by Mendelian Randomization (MR) methods. MR-like methods that discover a causal relationship between two gene trait variables using a genetic variant associated with both genes in a population include CIT [3] (and its precursor [4]), Findr [5], Trigger [6], and MRPC [7–9]. These methods utilize the associated genetic variant (single nucleotide polymorphism or SNP) as an instrumental variable to control for confounding between the observed genes using only observational data.

Despite research spanning two decades on MR methods for gene-gene causal discovery mentioned above, a limitation of most methods is their assumption of a linear relationship between the two genes or clinical traits being tested. Many trait pairs could significantly deviate from linearity (e.g., 84% of all tested pairs in the UK BioBank data [10]), and periodically changing genes (according to the circadian rhythm) could also exhibit nonlinear relationships [11]. A few studies have tried to incorporate nonlinearity [10,12,13], but they assume a piecewise linear or polynomial relation between the traits. Hence, there is a need for a more general nonlinear causal discovery method.

This work advances causal discovery research by proposing a new method NLCD for NonLinear Causal Discovery. NLCD employs conditional importance scoring of features in a general nonlinear regression model, in order to generalize a key conditional independence test of causality from linear to nonlinear settings. Specifically, NLCD extends a linear causal discovery method, CIT, by conducting a series of permutation-based statistical tests of causality in the nonlinear setting. The maximum p-value from these tests helps decide if data on a given triplet (two gene trait variables and a third instrumental variable) supports causality vs. independence between the traits. NLCD can flexibly work with any nonlinear regression model (including Kernel Ridge Regression, Support Vector Regression, or Artificial Neural Network models).

NLCD also differs from the broader ML (machine learning) based causal discovery literature in scope and assumptions. Score-based continuous optimization learners of the causal DAG (directed acyclic graph) structure - NOTEARS (Non-combinatorial Optimization via Trace Exponential and Augmented lagRangian for Structure learning) [14,15], GraN-DAG (Gradient-Based Neural DAG) [16], and DAG-GNN (DAG structure learning with Graph Neural Networks) [17] - jointly recover an *n*-variable DAG under causal sufficiency (no hidden confounders), an assumption typically violated in observational gene expression data. Constraint-based methods, such as the PC (Peter-Clark) algorithm with kernel-based conditional independence tests [18], can only learn a causal structure up to its Markov equivalence class from purely observational data, and therefore cannot identify direction in the confounded bivariate case. In the bivariate setting, functional causal models such as the additive noise model [19] and the post-nonlinear model [20] resolve direction from *A*,*B* alone but explicitly require the absence of a hidden confounder. There are ML-based algorithms that provide provable statistical and/or computational guarantees on nonlinear causal discovery from observational data under certain restrictive assumptions: DeFuSE (Deconfounded Functional Structure Estimation) [21] learns a nonlinear DAG under a sublinear growth assumption of the functional causal relationships, and an assumption that confounding manifests as correlated linear Gaussian errors, thereby allowing separation of nonlinear causal effects from linear confounding effects; and the NPVAR algorithm of Gao et al. [22] learns a model-free nonparametric (including nonlinear) causal DAG in polynomial time but under the restrictive assumptions of equal residual variances (i.e., conditional variance of a variable given its parents in the DAG are the same across all variables) and causal sufficiency (no unmeasured/hidden confounders). Rather than ruling out hidden confounding by assumption or adjusting it away under parametric constraints as done in the methods mentioned above, NLCD uses a genetic instrument to test the conditional-independence implications of an unconfounded causal model, so that confounded triplets are rejected rather than oriented. Similar to other genetic instrument based methods like Trigger and CIT, an oriented per-triplet call by NLCD certifies the absence of confounding, as per the causal equivalence theorem [6].

In benchmarks comprising simulated triplets, NLCD showed better performance in identifying nonlinear causal relations (for example, Average AUPRC was 0.72/0.57/0.51/0.71 in the order for NLCD/CIT/Findr/MRPC on a nonlinear sawtooth-type dataset). When benchmarked on a nonlinear subset of a yeast genomic dataset, NLCD’s average performance in recovering known causal relations involving transcription factors was comparable to CIT and better than Findr and MRPC. Application of NLCD to a human multi-tissue genomic dataset (called the Genotype-Tissue Expression or GTEx dataset) revealed a few causal gene pairs that are active in the muscle tissue, and highlighted the difficulty and hence the care that should be taken in filtering the discovered causal relations, as well as advantages of a data-driven approach to discover *in vivo* human causal gene networks.

## 2. Methods

### 2.1. Triplet-based causal discovery problem

**Fig 1:**
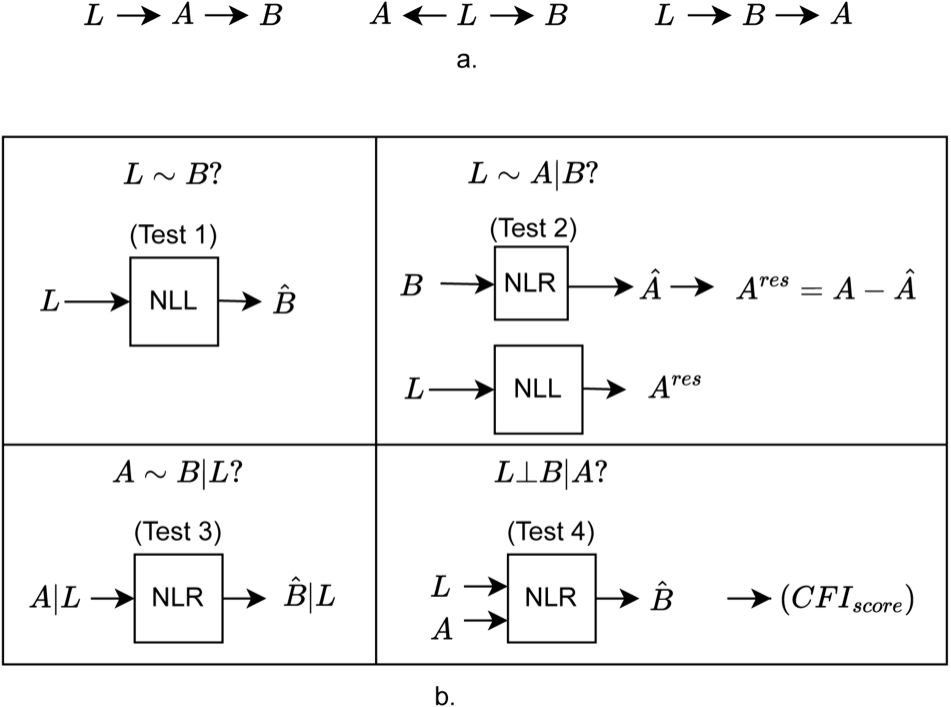
Schematic of our NLCD method. **a.** Given observational data on a genotype variable *L* and two gene expression variables *A* and *B* (measured across the same set of individuals), our method NLCD classifies the triplet (*L*,*A*,*B*) as following a causal (left), reactive (right), or independent (middle) model. **b.** To test if a triplet is causal, NLCD conducts four statistical tests: an association test (Test 1), two conditional association tests (Tests 2-3), and a key conditional independence test (Test 4). NLCD’s main contributions include the use of NonLinear Regression (NLR) modeling to perform three relevant tests and the development of a Conditional Feature Importance (CFI) score to implement Test 4. NLL stands for the Negative Log Likelihood test statistic.

We are interested in the problem of testing whether a causal relationship exists between two trait variables *A* and *B* (e.g., two gene expression traits focused in this work; clinical traits can also be considered), when there exists a third instrumental variable *L* (e.g., a SNP capturing the genetic variation occurring at a nucleotide in the genome). Together, these three variables are called a triplet (see Fig 1A).

**Input:** The triplet (*L*,*A*,*B*) can be thought of as three random variables that are observed in a set of *n* individuals, with the individuals randomly sampled from a population of interest. This observational (also referred to sometimes as observed or training) data on the triplet forms the input, and denoted as $D_{T}=\{(L_{1},A_{1},B_{1}),(L_{2},A_{2},B_{2}),...,(L_{t},A_{t},B_{t}),...,(L_{n},A_{n},B_{n})\}$ (see Table A in S1 Text). Note that *L* is a discrete random variable representing a genetic factor (e.g., a SNP), and can have values 0 or 1 for a haploid individual (e.g., yeast segregant) or values 0, 1 or 2 for a diploid individual (e.g., human). *A* and *B* are continuous random variables representing a pair of gene expression or any other traits.

**Output:** The expected output is to call that the triplet follows one of the three models, causal, reactive, or independent (see Fig 1A), based on the input observational data on the triplet. Output includes p-values associated with the call. Output can also be “No call” if evidence is inconsistent or inconclusive.

**MR-like assumptions/requirements:** Any method that solves the above causal discovery problem need to make certain assumptions. We are interested in only a class of methods that make the key assumption that *L* is ”randomized”, i.e., if each individual’s *L* value is randomly set (to one of its possible alleles) before and independent of the individual’s *A* and *B* (gene expression) values. As explained in Section 1.1 in S1 Text Background on underlying theorem and model, this assumption is one of the standard assumptions in MR as it is supported by randomness in biological processes such as reproductive cell formation; and enables development of methods that use *L* as a valid instrument or “causal anchor” to infer the causal relation between *A* and *B* based on an established causal equivalence theorem [3,6].

For convenience, certain methods make a few additional assumptions about the input triplet that can be easily verified using the triplet’s data. We can assume that the two genes in the triplet are correlated (i.e., A~B), since only correlated genes need be tested for causation. For *L* to be an instrument, we can also assume that *L* is correlated to both *A* and *B*. If the allelic variation at *L* is associated with the expression variation of a gene *G* across individuals, then this correlated (L~G) pair is called an expression Quantitative Trait Loci (eQTL) relation, with *L* being the eQTL SNP and *G* being the eQTL gene in the pair. Methods can filter triplets to focus only on triplets where *L* is an eQTL SNP for both *A* and *B*.

### 2.2. NLCD algorithm

Our NLCD method solves the triplet-based causal discovery problem by generalizing existing approaches, which typically assume a linear relationship between *A* and *B*, to the nonlinear setting. NLCD’s key contributions are to use a NonLinear Regression (NLR) model to capture the nonlinear causal relation between *A* and *B*, and to develop conditional feature importance (CFI) and related measures to properly realize four statistical tests that establish causality as per the causal equivalence theorem. We describe these contributions after providing some preliminaries. Note that NLCD can also discover linear causal links, as linearity is a special case encompassed by the broader nonlinear relations modeled by NLR.

#### 2.2.1. Preliminaries.

To clarify the context for our NLR models and statistical tests of the causal equivalence theorem (Fig 1), we explicitly provide the joint distribution of the three models of interest. For a random variable *X*, let $X\sim 𝒩(\mu ,\sigma^{2})$ denote that *X* is sampled from or follows a univariate Gaussian distribution with mean μ and variance $\sigma^{2}$, and $𝒩(x;\mu ,\sigma^{2})$ denote the probability density function of this Gaussian random variable *X* evaluated at *x* (i.e., $𝒩(x;\mu ,\sigma^{2}):=\frac{1}{\sigma \sqrt{2\pi }}\exp\{-\frac{(x-\mu {)}^{2}}{2\sigma^{2}}\}$).

Following standard probabilistic graphical model framework [23], the joint distribution for the causal model L→A→B is given by P(A,B,L)=P(L) P(A∣L) P(B∣A), reactive model is similarly factorized, and (conditional) independence model A←L→B is given by P(A,B,L)=P(L) P(A∣L) P(B∣L). Here *P* denotes the appropriate probability mass or density function. For instance, the prior *P*(*L*) is the probability mass function of a Bernoulli distribution (for haploids) or multinoulli/categorical distribution (for diploids). The conditional $P(A|L)=𝒩(A;\mu_{L},\sigma_{L}^{2})$, where the mean and variance parameters depend on the value of *L*. P(B∣L) is similarly defined but using its own set of parameter values (denoted $\left\{\mu_{L}^{\left(B\right)},\sigma_{L}^{2(B)}\right\}$, or simply $\left\{\mu_{L},\sigma_{L}^{2}\right\}$ by abusing notation when the context is clear about gene *B*). To complete the specification of the joint distribution, we need to specify the conditional B∣A as described next.

#### 2.2.2. NonLinear Regression (NLR) modeling.

NLCD captures nonlinear relationship via the conditional distribution $\left(B|A)\sim 𝒩(f(A),\sigma^{2}\right)$, where *f*(.) is a nonlinear function of its argument. While testing for conditional independence, we also need another conditional distribution, which we model as $\left(B|L,A)\sim 𝒩(g(L,A),{\sigma^{\prime }}^{2}\right)$, where *g*(.,.) is another nonlinear function of its arguments.

To learn these nonlinear functions *f*(.) and *g*(.) from data, we employ NLR. Simple NLR models suffice due to *L*, *A*, and *B* each being a one-dimensional random variable. We specifically use the following models from the scikit-learn library [24]: Artificial Neural Network (ANN) with one hidden layer, Support Vector Regression (SVR) with radial basis function (RBF) as the kernel, and Kernel Ridge Regression (KRR) with RBF as the kernel. The relevant scikit-learn functions were called as detailed in Section 1.2 in S1 Text Specifications of the nonlinear models used. Note that the learnt *f*(.) and *g*(.) may be linear when the data supports it, as nonlinear functions include linear functions as a special case.

#### 2.2.3. Conditional feature importance (CFI) score.

To realize a conditional independence test that is needed to discover causality, we need a corresponding statistic that captures the strength of the relation between two variables (*L* and *B* in our case), conditional on a third variable (*A* in our case). We propose a CFI score for this purpose; it is inspired by the conditional predictive impact measure from an earlier work [25].

Consider an NLR model to predict *B* from *L* and *A* (denoted B∣L,A), and let it be trained on the triplet’s data $D_{T}=\{(L_{t},A_{t},B_{t}){\}}_{t=1}^{n}$. Let ${\hat{Y}}_{L=\ell ,A_{t}}$ be the prediction of this model when L=ℓ and $A=A_{t}$. Let


$$\begin{array}{c} h_{j,k}(t)=\min\{P(L=j|A_{t}),P(L=k|A_{t})\} \end{array}$$

where j,k∈{0,1} for haploids and j,k∈{0,1,2} for diploids (and the relevant probabilities calculated using Bayes’ theorem as shown in Section 1.3 in S1 Text Calculation of probabilities for CFI score). Our proposed CFI score for a dataset involving haploids is then:


$$\begin{array}{c} CFI(D_{T})=\frac{1}{n}\sum_{t=1}^{n}\left(\left|{\hat{Y}}_{L=0,A_{t}}-{\hat{Y}}_{L=1,A_{t}}\right|h_{0,1}(t)\right), \end{array}$$

and diploids is:


$$\begin{array}{c} CFI(D_{T})=\frac{1}{n}\sum_{t=1}^{n}\left(\frac{1}{3}\sum_{0\leq i<j\leq 2}\left|{\hat{Y}}_{L=i,A_{t}}-{\hat{Y}}_{L=j,A_{t}}\right|h_{i,j}(t)\right). \end{array}$$

We now provide rationale for the haploid CFI score, as the diploid CFI’s rationale is similar. The first term in the summand of haploid CFI quantifies the importance of feature *L* conditional on feature $A=A_{t}$ in an NLR model for predicting *B* (and thus helps test L⟂B∣A). But this term is not trustable if ${\hat{Y}}_{L=0,A_{t}}$ or ${\hat{Y}}_{L=1,A_{t}}$ is not trustable – which is the case when the NLR model has never seen datapoints in the vicinity of $\left(L=0,A=A_{t}\right)$ or $\left(L=1,A=A_{t}\right)$ during training. Therefore, the second term in haploid CFI’s summand, *h*_0,1_(*t*), gives more weight to $A_{t}$ values for which both *L* = 0 and *L* = 1 are likely to be seen during training.

To provide further insight into CFI, we define a related “overlap score” for a dataset involving haploids as:


$$\begin{array}{c} OverlapScore(D_{T})=\frac{1}{n}\sum_{t=1}^{n}h_{0,1}(t), \end{array}$$

and diploids as:


$$\begin{array}{c} OverlapScore(D_{T})=\frac{1}{n}\sum_{t=1}^{n}\left(\frac{1}{3}\sum_{0\leq i<j\leq 2}h_{i,j}(t)\right). \end{array}$$

Intuitively, the haploid overlap score captures the overlap between the support of two gene expression distributions (A∣L=0 and A∣L=1) and CFI captures the difference in predictions among different values of *L* in the regions of overlap. Similar intuition also applies to the diploid overlap score and CFI.

Note that when the overlap score is low (e.g., due to a strong eQTL effect that nearly separates A across genotype strata), the CFI score is effectively estimated from a few samples in the overlap region, which reduces the power of NLCD’s conditional independence test. This is an inherent data-driven limitation, and can be diagnosed using a low overlap score before interpreting a CFI-based result.

#### 2.2.4. Realizing the four statistical tests of causality.

As mentioned earlier, NLCD is based on an established causal equivalence theorem [6], where a chain of three statistical tests was used to test for causality (see Section 1.1 in S1 Text Background on underlying theorem and model). We rely actually on a modified version of this theorem [3], where the tests were slightly changed by conditioning on different variables and a fourth test was added to improve empirical performance (of a linear causal discovery method CIT). Our method NLCD extends the implementation of these four statistical tests (shown below and also in Fig 1B) from the linear to nonlinear setting using the concepts of NLR modeling and CFI scoring:

(Test 1): L~B
(Test 2): L~A∣B
(Test 3): A~B∣L
(Test 4): L⟂B∣A (Conditional Independence Test)

Following the original CIT framework [3], each of the four tests above is designed to rule out a specific non-causal alternative for the triplet (*L*,*A*,*B*), so that the conjunction of all four conclusions is logically equivalent to the causal model L→A→B (under the assumption that *L* is a valid instrument). Test 1 (L~B) confirms that *L* has a marginal effect on the outcome *B* that is large enough to be detectable, without which causality cannot be tested. Test 2 (L~A∣B) confirms that *L*’s effect on the exposure *A* persists after conditioning on the outcome, ruling out the reverse-causation direction B→A in which case *A* would carry no *L*-information beyond what *B* already carries. Test 3 (A~B∣L) requires *A* and *B* to be dependent within at least one *L*-stratum, ruling out the (conditional) independence model A←L→B. Finally, Test 4 (L⟂B∣A)—the only conditional *independence* test—requires *L*’s effect on *B* to be fully mediated by *A*, thereby ruling out a direct L→B edge (horizontal pleiotropy or unmodeled confounding). Each of these four tests is formulated as a hypothesis test with an explicit null (*H*_0_) and alternative (*H*_1_), stated within the corresponding test’s description below. Only when all four alternative hypotheses are supported by the data do we conclude L→A→B; the formal $H_{0}/H_{1}$ statements and a precise intersection–union combining rule are given below.

NLCD calculates the empirical p-value for each test using permutation testing. In detail, we calculate the test statistic on the actual observed data and several permuted datasets (with permutation appropriately done to make the permuted data conform to the null model). The empirical p-value is then estimated using the number of permutations (*m*) in which the statistic is as extreme as or more extreme than the statistic calculated on the actual observed data, as follows. If *M* is the total number of permutations (default *M* = 500), the estimated empirical p-value is given by $\frac{m+1}{M+2}$. The pseudo counts of +1 and +2 in this formula handle cases where *m* = 0. We now describe the statistic and permutation scheme used by NLCD in its four tests.

• **Test 1**: L~B

This is a test of the association between *L* and *B*. Formally, we test


$$\begin{array}{c} H_{0}^{\left(1\right)}:L⟂B\;\text{vs.}\;H_{1}^{\left(1\right)}:L\text{⧸}⟂B. \end{array}$$

Under the causal model L→A→B, *L* is marginally associated with *B* through the mediator *A*, so the causal model implies the alternative hypothesis $H_{1}^{\left(1\right)}$; a triplet for which the null hypothesis $H_{0}^{\left(1\right)}$ cannot be rejected is filtered out. Regardless of the underlying true model generating the data, we assume for this test that $\left(B|L=\ell )\sim 𝒩(\mu_{\ell },\sigma_{\ell }^{2}\right)$. Let $n_{\ell }$ be the number of training datapoints with genotype value ℓ, i.e., $n_{\ell }:=|\{t\in \{1,...,n\}:L_{t}=\ell \}|$. Then, we use the training data to compute the maximum likelihood estimates (MLEs) of (B∣L=ℓ) as:


$$\begin{array}{ccc} {\hat{\mu }}_{\ell } & = & \frac{1}{n_{\ell }}\sum_{t:L_{t}=\ell }B_{t}\text{, and}\\ {{\hat{\sigma }}_{\ell }}^{2} & = & \frac{1}{n_{\ell }}\sum_{t:L_{t}=\ell }(B_{t}-{\hat{\mu }}_{\ell }{)}^{2}; \end{array}$$

and use these MLEs computed for each possible value of ℓ to compute the Negative Log Likelihood (NLL) loss (i.e., observed test statistic) as:


$$\begin{array}{c} NLL=-\sum_{t=1}^{n}\ln\left(𝒩\left(B_{t}\;;\;{\hat{\mu }}_{L_{t}}\;,\;{{\hat{\sigma }}_{L_{t}}}^{2}\right)\right). \end{array}$$

Next, we randomly permute the *B* trait (i.e., the vector $\{B_{t}{\}}_{t=1}^{n}$) to break any association with *L*, and recompute the MLEs and NLL loss on this permuted dataset – this permutation-based NLL statistic is expected to be higher than the observed statistic if *L* is truly associated with *B*. Finally, repeating this process for *M* permutations and comparing the resulting statistics with the observed statistic as explained above yields the empirical p-value for this test.

• **Test 2**: L~A∣B

We test the association of *L* and *A* given *B*. Formally,


$$\begin{array}{c} H_{0}^{\left(2\right)}:L⟂A|B\;\text{vs.}\;H_{1}^{\left(2\right)}:L\text{⧸}⟂A|B. \end{array}$$

Under the causal model L→A→B, conditioning on the descendant *B* does not make *L* independent from *A*, so *L* remains associated with *A* given *B*; the causal model therefore implies $H_{1}^{\left(2\right)}$. We use the training data to learn a NLR model to predict *A* from *B*, i.e., learn a nonlinear function *f*(.) such that A≊f(B). Then, for the *t*-th training datapoint, we have the prediction ${\hat{A}}_{t}=f(B_{t})$, and residual $A_{t}^{res}=A_{t}-{\hat{A}}_{t}$. Let $A^{res}$ denote the residual variable (and also the vector $\{A_{t}^{res}{\}}_{t=1}^{n}$). We finally *t*est $L\sim A^{res}$ using the same association test described above (Test 1), but with $A^{res}$ used in place of *B* to get a p-value for association between *L* and (A∣B).

• **Test 3**: A~B∣L

This test detects association between *A* and *B* in any strata of *L*. Per stratum L=ℓ, we test


$$\begin{array}{c} H_{0}^{\left(3,\ell \right)}:A⟂B|L=\ell \;\text{vs.}\;H_{1}^{\left(3,\ell \right)}:A\text{⧸}⟂B|L=\ell , \end{array}$$

and we aggregate across strata by defining


$$\begin{array}{c} H_{0}^{\left(3\right)}:\underset{\ell }{⋂}H_{0}^{\left(3,\ell \right)}\;\text{vs.}\;H_{1}^{\left(3\right)}:\underset{\ell }{⋃}H_{1}^{\left(3,\ell \right)}, \end{array}$$

i.e., *A* and *B* are associated within at least one *L*-stratum. Under the causal model L→A→B, the direct edge A→B induces conditional dependence in at least one *L*-stratum, so the causal model implies $H_{1}^{\left(3\right)}$. Operationally we estimate the minimum p-value across strata to implement this union alternative: to do so, we obtain a p-value of association between *A* and *B* for each possible value of *L* as explained next, and take the minimum of the obtained p-values as the final p-value of Test 3. For any given value ℓ of *L* (with ℓ∈{0,1} in case of haploid and ℓ∈{0,1,2} for diploid), we use the subset of training datapoints with $L_{t}=\ell$ to fit a NLR model to predict *B* from *A*, and take the associated mean square error (MSE) loss shown below as the observed test statistic:


$$\begin{array}{c} {MSE}_{\ell }=\frac{1}{n_{\ell }}\sum_{t:L_{t}=\ell }{\left(B_{t}-{\hat{B}}_{t}\right)}^{2}, \end{array}$$

where ${\hat{B}}_{t}$ is the model prediction based on input $A_{t}$. Next, as in Test 1, we independently and randomly permute the *B* trait but within the stratum defined by L=ℓ (i.e., the vector $\left\{B_{t}:L_{t}=\ell \right\}$) to break any association with *A* within this strata, and refit the NLR model again within strata ℓ and recompute the ${MSE}_{\ell }$ loss (which is expected to be higher than the observed statistic if there is true association). Repeating this process *M* times and comparing the resulting permutation-based statistics with the observed statistic yields the empirical p-value of A~B given L=ℓ.

• **Test 4 (Conditional independence test)**: L⟂B∣A

Test 4 is the one test whose null and alternative are framed opposite to the classical association-testing convention (the equivalence testing framework), because the causal model L→A→B requires *L* independent of *B* conditioned on *A*:


$$\begin{array}{c} H_{0}^{\left(4\right)}:L\text{⧸}⟂B|A\;\text{vs.}\;H_{1}^{\left(4\right)}:L⟂B|A. \end{array}$$

Thus the causal model implies $H_{1}^{\left(4\right)}$ (conditional independence), and rejecting $H_{0}^{\left(4\right)}$ in favor of $H_{1}^{\left(4\right)}$ at level α constitutes the key causal evidence contributed by this test. The stratified permutation scheme described below constructs a null distribution that conforms to $H_{0}^{\left(4\right)}$ (i.e., preserves *L*–*B* dependence while destroying *A*–*B* dependence within each *L*-stratum), so that a small observed CFI relative to this null distribution yields a small empirical p-value, i.e., evidence for $H_{1}^{\left(4\right)}$.

To test this conditional independence, we first fit an NLR model, with *L* and *A* as inputs and *B* as output, to the training data; and use the associated CFI score as the observed test statistic (see Section 2.2.3 Conditional Feature Importance (CFI) score on the derivation of CFI, and how CFI is a relevant test statistic quantifying the conditional dependence between *L* and *B* given *A*).

Next, we seek a permutation scheme for shuffling the observed data to match a stringent null model—one that eliminates the conditional independence of interest while preserving all other inter-variable relationships as much as possible. One such scheme is a random permutation of *B* stratified by *L*. This permutation breaks any dependence between *B* and *A* within each stratum defined by *L*, thereby preserving any marginal dependence between *B* and *L*, and importantly enforcing the null model L⧸⟂B∣A [3]. Note that the null model cannot be enforced if there is no marginal B~L dependence in the observed data; however this is not a concern as Test 1 can filter out such triplets. The process to compute a permutation-based CFI comprises these steps: independently and randomly permute the values of *B* within each stratum defined by *L* to obtain the permuted vector $B_{stratperm}$, fit an NLR model to the training data with *B* replaced by $B_{stratperm}$, and compute the associated CFI (which is expected to be greater than the observed CFI if *B* is truly conditionally independent of *L* given *A*). As before, repeating this process *M* times and comparing the resulting statistics with the observed statistic yields the empirical p-value for Test 4.

**Obtaining the final p-value:** Across the four tests, the causal model L→A→B corresponds to the composite alternative


$$\begin{array}{c} H_{1}\;=\;H_{1}^{\left(1\right)}\cap H_{1}^{\left(2\right)}\cap H_{1}^{\left(3\right)}\cap H_{1}^{\left(4\right)}, \end{array}$$

i.e., *all four* individual alternatives must hold simultaneously. The corresponding composite null is the union


$$\begin{array}{c} H_{0}\;=\;H_{0}^{\left(1\right)}\cup H_{0}^{\left(2\right)}\cup H_{0}^{\left(3\right)}\cup H_{0}^{\left(4\right)}. \end{array}$$

This is precisely the setting of an *intersection-union* test framework [26]: the composite null *H*_0_ is rejected at level α if and only if *every* individual null $H_{0}^{\left(i\right)}$ is rejected at level α. Equivalently, letting $p_{i}$ denote the empirical p-value of test *i*, and following the intersection-union test framework, we take the maximum of the p-values from the four tests above to get a single p-value,


$$\begin{array}{c} p_{final}\;=\;\max(p_{1},p_{2},p_{3},p_{4}). \end{array}$$

On this final p-value, we apply a suitable cutoff (say a significance level of 0.05) to decide if the triplet is causal (L→A→B) or not. The intersection-union rule controls the Type I error of the composite test at level α by construction [26]: rejecting *H*_0_ requires each $p_{i}\leq \alpha$, which is equivalent to $p_{final}=\max_{i}p_{i}\leq \alpha$. An empirical assessment of Type I error control is provided in Section 2.1 in S1 Text Assessing NLCD’s control of Type I error.

#### 2.2.5. Making the final call.

If we know *a priori* that *A* is the potential regulator/exposure variable and *B* is the potential target/outcome variable, then we need to apply NLCD tests only in the forward direction L→A→B and compare it to a cutoff (say 0.05) as discussed above to call whether the triplet is causal or not. We make NLCD calls in this fashion when analyzing simulated and yeast benchmark datasets.

If the causal direction is not known *a priori* like in the human dataset, then the same procedure discussed above to calculate the final p-value in the forward direction L→A→B is also used to calculate a final p-value in the reverse direction L→B→A by swapping the inputs *A* and *B* of NLCD’s tests. Then we use these two p-values, along with a cutoff (significance level of 0.05 say), to make the final call about which model a triplet’s data supports. Specifically as in CIT [3], we call a triplet as:

- causal if forward, but not reverse, p-value is less than 0.05.
- reactive if reverse, but not forward, p-value is less than 0.05.
- independent if both p-values are greater than 0.05.
- inconclusive (no call) if both p-values are less than 0.05.

#### 2.2.6. Optional postprocessing step.

Using *M* permutations, the resolution of the empirical p-value of our tests and the smallest achievable empirical p-value are both 1/(*M* + 2). For applications requiring higher resolution of small p-values, we can increase *M*; but relying only on increased permutations has a computational cost. So we employ two steps: increase *M* to a modest 1000 (from the default 500), and postprocess the resulting statistics using a method called EPEPT (Enhanced P-value Estimator for Permutation Tests) [27,28]. EPEPT employs a generalized Pareto distribution to approximately model the tail of the distribution of permutation-based test statistics. This semi-parametric approach results in higher resolution of small p-values using fewer permutations than the standard non-parametric empirical p-value approach. We downloaded a MATLAB code that implements EPEPT from https://github.com/IlyaLab/epept, and used it to improve the resolution of p-values obtained from applying NLCD to the human GTEx dataset (note that for other datasets considered in this study, the default resolution of p-values was sufficient).

### 2.3. Generation of simulated datasets

To test whether our method’s p-values are sufficiently calibrated to distinguish data from causal vs. independent triplets, we simulated linear and nonlinear datasets pertaining to causal triplets and another dataset pertaining to independent triplets. These datasets were generated under two settings: one where the variances of trait *A* across different strata of *L* are equal, and another where these variances are unequal.

#### 2.3.1. Generation of data with equal variance.

Here, the variances of *A* conditioned on different values of *L* are the same. Specifically, we simulated only triplets with haploid genotypes that satisfy:


$$\begin{array}{c} Var(A|L=0)\;=\;Var(A|L=1). \end{array}$$

Under this setting, we generated five types of simulated datasets: linear, sine, sawtooth, parabola, and independent. The first four datasets follow the causal model L→A→B with the direct A→B relation following a linear model for the linear simulated dataset and a nonlinear relation such as sine, sawtooth or parabola for the other datasets. The independent dataset follows the independent A←L→B model where there is no direct A−B relation.

**Fig 2:**
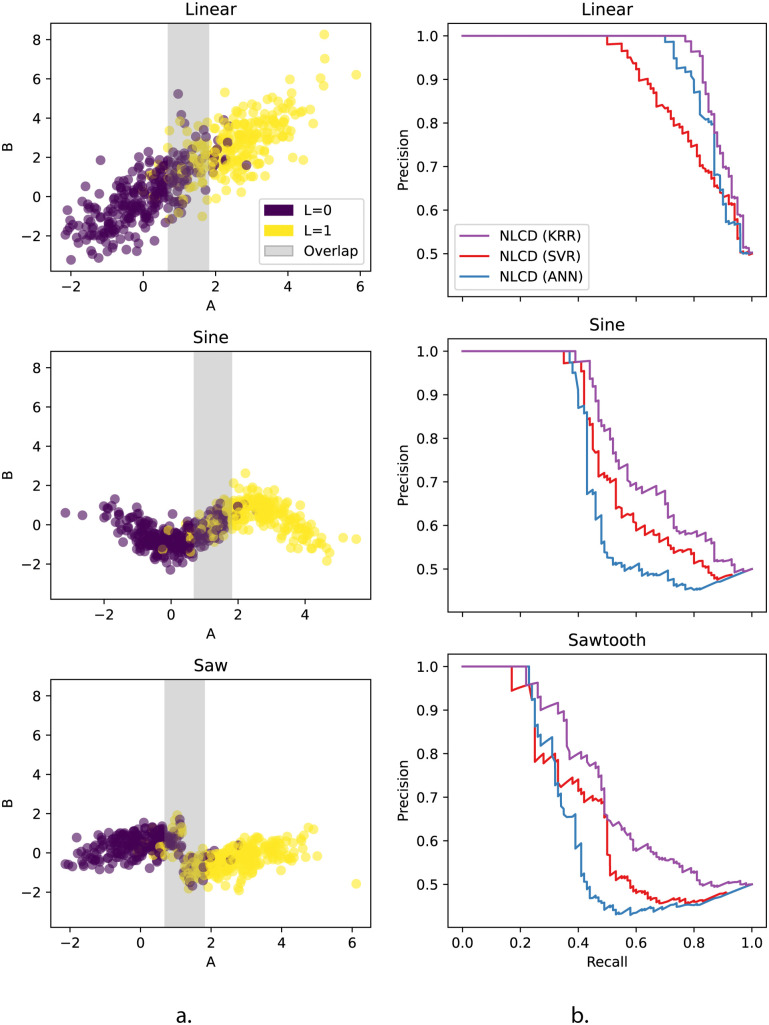
NLCD on simulated benchmark datasets. **a.** Example plots of the simulated *A* and *B* genes illustrate different types of gene-gene relationships, linear and nonlinear (sine and sawtooth). Shaded grey marks the overlap region between the two gene expression distributions: (A∣L=0) and (A∣L=1), with endpoints of this overlap region obtained by setting P(L=0∣A) to 0.2 and to 0.8 and solving for *A* (see formula derived in Section 1.4 in S1 Text). **b.** Performance of NLCD on benchmarks, each comprising data simulated on 100 causal vs. 100 independent triplet models, on a binary (causal vs. independent) classification task is reported as Precision-Recall (PR) curves. Sample size of *n* = 500 is used here.

To elaborate each dataset further, let’s consider a sine dataset as an example. This dataset comprises 100 different triplets, each of which contains simulated data (*L*,*A*,*B* measurements) of *n* individuals/samples. The 100 triplets differ from each other based on their parameter values. There are 10, 5, and 2 distinct values that μ, $\sigma_{1}$, and $\sigma_{2}$ parameters can take respectively, resulting in 10×5×2=100 parameter combinations. For each such combination corresponding to a triplet, its data is generated by independently sampling *n* times from the distributions listed below. The sawtooth dataset is also generated from the same 100 parameter combinations as the sine dataset as shown below. The linear, parabola, or independent dataset is generated using the parameters $\beta_{1}$ and $\beta_{2}$ (with each parameter taking 10 distinct values, resulting in 10×10=100 parameter combinations or triplets; see below). Data plots of example triplets are in Fig 2A.


$$\begin{array}{cc} & L\sim Bernoulli(0.5)\\ & \beta_{1}\in \{0.1,0.5,1,1.5,2,2.5,3,4,5,6\}\\ & \beta_{2}\in \{0.1,0.5,1,1.5,2,2.5,3,4,5,6\}\\ & A=\beta_{1}L+\epsilon_{1}\\ & B=\beta_{2}f(A)+\epsilon_{2}\\ & \epsilon_{1}\sim 𝒩(0,1)\;\epsilon_{2}\sim 𝒩(0,1)\\ & f(A)=A\;\left(Linear\right)\\ & f(A)=A^{2}\;\left(Parabola\right)\\ & f(A)=L\;\left(Independent\right) \end{array}$$

$$\begin{array}{cc} & L\sim Bernoulli(0.5)\\ & \mu \in \{0.1,0.5,1,1.5,2,2.5,3,4,5,6\}\\ & \sigma_{1}\in \{0.2,0.4,0.6,0.8,1\}\\ & \sigma_{2}\in \{0.5,1\}\\ & A=\mu L+\epsilon_{1}\\ & B=g\left(\frac{2\pi A}{8\sigma_{1}}-\frac{\pi }{2}\right)+\epsilon_{2}\\ & \epsilon_{1}\sim 𝒩(0,1)\;\epsilon_{2}\sim 𝒩(0,\sigma_{2})\\ & g(.)=sin(.)\;\left(Sine\right)\\ & g(.)=sawtooth(.)\;\left(Sawtooth\right) \end{array}$$

Functions sin(.) and sawtooth(.), from Python’s numpy and scipy libraries respectively, produce the corresponding waveforms with period 2π. For instance, sawtooth(.) produces a waveform that increases linearly from −1 to 1 over the interval [0,2π), and jumps back to −1 at the end of each period.

#### 2.3.2. Generation of data with unequal variance.

Here, the variances of *A* conditioned on different values of *L* are unequal. Specifically, we simulated only triplets with haploid genotypes that satisfy:


$$\begin{array}{c} Var(A|L=0)\;\neq \;Var(A|L=1). \end{array}$$

We generated two datasets, linear with unequal trait variance and parabola with unequal trait variance, using the parameter list $\beta_{1}$, $\beta_{2}$, and α (each with 5, 5, and 4 distinct values respectively, resulting again in 100 parameter combinations). The simulation process is shown below. Besides simulating triplets with unequal conditional trait variances $\beta_{1}\neq \beta_{2}$, this process also simulates a smaller fraction of triplets with $\beta_{1}=\beta_{2}$.


$$\begin{array}{cc} & L\sim Bernoulli(0.5)\\ & \beta_{1}\in \{1,2,3,4,5\}\\ & \beta_{2}\in \{1,2,3,4,5\}\\ & \alpha \in \{1,2,3,4\}\\ & A=\alpha L+L\epsilon_{1}+(1-L)\epsilon_{2}\\ & B=h(A)+\epsilon_{3}\\ & \epsilon_{1}\sim 𝒩(0,\beta_{1})\;\epsilon_{2}\sim 𝒩(0,\beta_{2})\;\epsilon_{3}\sim 𝒩(0,1)\\ & h(A)=A\;\left(Linear,\;unequal\;variance\right)\\ & h(A)=A^{2}\;\left(Parabola,\;unequal\;variance\right) \end{array}$$

### 2.4. Yeast benchmarking methodology

We evaluated NLCD on a real-world yeast dataset using the benchmarking methodology described in this section.

#### 2.4.1. Yeast data/triplets setup.

**Yeast genomic data and its preprocessing**: The yeast genomic data, comprising genotype information, transcriptomic (gene expression) profiles, and covariates of 1012 yeast segregants (derived from a cross of two yeast strains) were obtained from an earlier published study [29]. Albert et al. had adjusted the gene expression data for the two covariates, experimental batch and a growth-rate covariate, using a linear regression model to remove potential technical confounding effects prior to eQTL mapping. We performed a similar adjustment before further analysis. From the eQTLs detected and reported by Albert et al., we retrieved a list of *cis*-eQTL genes, and the strongest *cis*-eQTL variant for each such gene to select the instrumental variable for the causal discovery methods. For yeast gene annotations, yeast Ensembl release 109 was used.

**Ground-truth TF** → **TG data**: The ground-truth yeast regulatory relations from transcription factors (TFs) to target genes (TGs) were obtained from the work of [30] (https://github.com/michoel-lab/FindrCausalNetworkInferenceOnYeast), who in turn obtained it from YEASTRACT+ [31], a curated repository of global transcriptional regulation in yeast species. This repository contains two kinds of experimental evidence for causal TF→TG relations: *DNA binding* evidence (from chromatin immunoprecipitation (ChIP) related experiments) and *expression* evidence (from experimentally-observed expression changes in TGs upon perturbation of a TF). We used ground-truth regulatory relations supported by both DNA binding and gene expression evidence. To derive a subset of these ground-truth relations that are amenable to discovery from Albert et al. data by MR-like causal discovery methods, we retained only: (i) TFs that were identified as *cis*-eQTL genes in the Albert et al. study, and (ii) TGs whose expression was available in Albert et al.’s transcriptomic data. The final ground-truth matrix had 80 TFs along rows, 3422 TGs along columns, and with each matrix entry being either 1 to indicate causal regulation or 0 to indicate non-regulation (of the corresponding TF-TG pair).

**Assembling the benchmark of yeast triplets**: For each TF *A* in the final ground-truth matrix, we assembled triplets of the form (*L*,*A*,*B*) by selecting its strongest *cis*-eQTL SNP *L* (i.e., the *cis*-eQTL variant with the highest absolute correlation coefficient to the *A* gene as reported in Albert et al.), and any gene *B* that is associated with *L* at FDR (False Discovery Rate) 20% in Albert et al.’s data. There are 3422 potential *B* genes (TGs or columns of the final ground-truth matrix mentioned above); so to obtain associations at FDR 20%, we test for association between the SNP *L* and each potential *B* gene using Wilcoxon unpaired test, subject the resulting 3422 p-values to Benjamini-Hochberg multiple testing correction, and select *B* genes with corrected p-value ≤0.20 (note that this procedure excludes from analysis any self regulation where *B* is the same as *A*). Assembling triplets using this procedure ensures that *L* is a valid instrumental variable associated with both *A* and *B* genes. Note also that this procedure resulted in certain *A* genes with no corresponding *B* genes—such *A* genes are excluded from further analysis.

An assembled triplet (*L*,*A*,*B*) is considered (truly) causal if A→B is 1 in the ground-truth TF → TG matrix, and independent (non-causal) otherwise. For each *cis*-eQTL gene *A*, we found that the independent triplets involving *A* outnumbered the causal triplets involving *A*; so we randomly downsampled the independent triplets to create a balanced dataset with equal number of causal vs. independent triplets for the *A* gene. The resulting *yeast benchmark* contained 1752 causal triplets (involving 66 unique *cis*-eQTL TFs and 1258 unique TGs) and 1752 independent triplets (involving 66 unique *cis*-eQTL TFs and 1395 unique TGs), and is used for all further analysis. Note that each triplet’s data has a sample size of 1012 (due to *L*,*A*,*B* being measured across 1012 yeast segregants).

#### 2.4.2. Yeast triplet subsets with different correlation strengths/types.

We create different subsets of the triplets in the yeast benchmark by applying cutoffs on measures of linear and nonlinear type correlation, and another measure on the extent of nonlinearity. The resulting high-quality subsets of ground-truth triplets exhibited nonlinearity to varying extents, and can hence be used to find the relative strengths and limitations of different causal discovery methods including ours.

**Correlation measures used**: We employed two correlation measures: Bias Corrected Mutual Information (BCMI) [32,33] to quantify the strength of any nonlinear association between two variables, and Spearman correlation coefficient (ρ) to quantify the strength of linear/monotonic relationship between the variables. In detail, BCMI is a modified (jackknife-based bias-corrected) estimator of mutual information, which in turn is a widely used measure of nonlinearity and non-monotonicity of association between two variables. We preferred Spearman over Pearson correlation (although Pearson directly measures the strength of a linear relationship, and Spearman captures monotonic relationships that include linear associations as a special case) because Spearman is more robust to outliers.

**Extent of nonlinearity (**δ): For each causal triplet in the yeast benchmark, we computed both BCMI and ρ between the genes in the triplet using Albert et al. data. We then used the resulting values across all causal yeast triplets to regress |ρ| on BCMI, thereby defining an expected relationship between the two measures. We define the extent of nonlinearity (δ) of a triplet as the shortest distance of its corresponding point from the regression line, focusing only on points below the regression line to identify nonlinear triplets (i.e., triplets whose |ρ| is smaller than expected from the regression, indicating that BCMI is relatively high).

**Creating correlation-based benchmark subsets**: We create different subsets of the yeast benchmark by subjecting its 1752 causal and 1752 independent triplets to different cutoffs (lower bounds) on either BCMI or |ρ|, and further subjecting its causal triplets alone to different cutoffs on δ. BCMI cutoffs are chosen from 0.05, 0.10, and 0.15; and the corresponding |ρ| cutoffs are the predicted |ρ| values for each of these BCMI cutoffs using the regression model described above. For each of these cutoff values, we selected triplets with BCMI (or |ρ|) at least the cutoff, so that they are sufficiently correlated and hence amenable for causal discovery by the evaluated methods. The result is a high-quality subset of the yeast benchmark, due to removal of several triplets with BCMI (or |ρ|) near zero (i.e., triplets whose genes are poorly or not correlated due to noise inherent in real-world data and ground truth not being perfect).

Cutoffs on the extent of nonlinearity, δ, are chosen from {0, 0.0125, 0.025, 0.0375, 0.05, 0.1}, such that applying a cutoff of 0 selects all points (causal triplets) below the regression line, 0.0125 selects all points below the regression line at a distance > 0.0125 from the line, and so on. These δ cutoffs yield different yeast benchmark subsets with varying degree of nonlinearity in the gene-gene association in the causal triplets. We do not subject independent triplets to the δ cutoff due to the assumption that the genes in an independent triplet are linked only via *L* and not directly to each other (and hence there is no nonlinear association between the genes on which the δ cutoff could be applied).

Just as in the full yeast benchmark, many benchmark subsets created above have more independent than causal triplets. For each such subset, we randomly downsampled the independent triplets to the same number as the causal triplets to ensure class balance (note that other subsets with fewer independent than causal triplets are left untouched). We repeat this process 10 times using different random seeds to obtain 10 different independent triplet lists. Combining each of these independent triplet lists (negative examples) with the causal triplets of the subset (positive examples) yields 10 different random versions of each yeast benchmark subset—performance measures like AUPRC of a benchmark subset are reported using their average and standard deviation across these 10 random versions.

### 2.5. Evaluation of causal discovery methods

We evaluated our NLCD and other existing state-of-the-art (SOTA) methods for causal discovery (CIT, Findr, MRPC) using a classification task and associated benchmark datasets described herein.

#### 2.5.1. Classification task and benchmark datasets.

**Classification task:** The binary classification task is to predict whether an input triplet is causal vs. independent. Specifically, the task is to classify whether an input triplet’s data, comprising its (*L*,*A*,*B*) measurements across *n* individuals, follows a causal or an independent model. We can evaluate a method performing this task using a benchmark dataset that combines data on a certain number of distinct causal triplets (positive class examples) with that of distinct independent triplets (negative class examples).

**Simulated benchmark datasets:** Each simulated benchmark dataset comprises a simulated dataset on 100 causal triplets and another on 100 independent triplets (see Section 2.3 Generation of simulated datasets). Recall that the linear, sine, or sawtooth simulated dataset comprises data on 100 distinct causal triplets, with each triplet’s data being of sample size *n* and generated using different parameter values of the underlying causal model (see Fig 2A). Similar to this simulated causal dataset, the simulated independent dataset comprises data on 100 different independent triplets generated using different parameter values of the independent model.

**Yeast benchmark datasets:** The full yeast benchmark dataset contains data on 1752 causal and 1752 independent triplets assembled as described above (see Section 2.4 Yeast benchmarking methodology). Also as described above (see Section 2.4.2 Yeast triplet subsets with different correlation strengths/types), we took nonlinear vs. linear subsets of the causal triplets and matched independent triplets to create different yeast benchmark subsets. We used these benchmark subsets to evaluate NLCD and SOTA methods on triplets with different degrees of nonlinear vs. linear type gene-gene association.

#### 2.5.2. Evaluation measure.

AUPRC, calculated using the average precision score, was used to evaluate the performance of the classification models. Though AUPRC can also be calculated using trapezoidal approximation, it involves linear interpolation which is not ideal in the precision-recall space. Average precision score avoids this issue and is also suited for skewed data [34,35].

#### 2.5.3 Causal discovery calls from evaluated methods.

The evaluated methods differ in how they are run on the benchmark triplets’ data (one triplet at a time separately, or on all triplets’ combined data at once), and how they make causality calls (based on applying cutoffs on causality p-values or probabilities). This section provides these details for each evaluated method. Note that the methods are run on each simulated dataset and on the full yeast benchmark dataset. We do not run the methods separately on each yeast benchmark subset; instead, we evaluate performance on these different subsets by extracting and analyzing the relevant causality calls obtained from the full yeast benchmark run.

**NLCD calls:** In our simulated and yeast benchmark datasets, we know the ground truth causal direction. So as discussed in Section 2.2.5 Making the final call, we apply NLCD tests on each triplet only in the forward L→A→B direction; and if the resulting p-value is less than a cutoff (say 0.05), we call the triplet causal, otherwise independent.

**CIT calls:** NLCD is an extension of the CIT framework to capture nonlinear relations, so CIT calls are also made the same way as NLCD calls above.

**Findr calls:** Given a sufficient number of input triplets, Findr [5] outputs the probability of a triplet to be causal based on the probability of three of the five component tests implemented in Findr as follows:


$$\begin{array}{c} P_{final}=\frac{1}{2}(P_{2}P_{5}+P_{4}). \end{array}$$

To estimate this probability for each triplet, the findr function findr.pij_gassist needs to be applied on the concatenated data of all triplets of interest through a single function call (and not as a separate function call per triplet), as findr needs statistics from a sufficient number of triplets following the null vs. alternate hypotheses to estimate these probabilities.

The concatenated data also needs to be organized in a certain fashion as three matrices [36] that we explain below, and the above findr function’s output probability matrix needs to be subsetted to extract the causal probabilities of the triplets of interest. More details are in Findr software’s documentation/manual and our code.

To run Findr on each simulated benchmark, we organized the *n* simulated observations on the *m* causal and independent triplets in the benchmark into 3 different matrices: $L_{mat}\in \{0,1{\}}^{m\times n},A_{mat}\in {\mathbb{R}}^{m\times n}$, and $B_{mat}\in {\mathbb{R}}^{m\times n}$. Here, $L_{mat}[i,j]$ is the (haploid) genotype of the $i^{th}$ triplet’s SNP *L* in the $j^{th}$ individual/sample, $A_{mat}[i,j]$ is the expression of the $i^{th}$ triplet’s *A* gene in the $j^{th}$ sample, and $B_{mat}[i,j]$ is also defined similarly.

To run Findr on the yeast benchmark, we organized the observed genomic data on the causal and independent triplets in the benchmark into three matrices, as done above for the simulated benchmark. In addition, we reordered the rows of these matrices to handle genes that overlap between the sets of *A* and *B* genes across all triplets. Specifically, these overlapping genes were placed first and in the same order in the reordered $A_{mat}$ and $B_{mat}$, followed by the other genes (which were also kept in the same order). The $L_{mat}$ was also reordered accordingly, such that its $i^{th}$ row corresponds to the genotype values of the strongest *cis*-eQTL/SNP of the $i^{th}$ gene (row) of $A_{mat}$.

**MRPC calls:** MRPC was run on each benchmark triplet separately, as this triplet-wise running performed competitively with other methods on the simulated benchmarks, and better than batch-wise running of MRPC on the yeast benchmark as detailed below. We ran MRPC on the data of a given triplet by following these recommendations from its authors: (i) organize data on the triplet into a table with columns in the order *L*, *A*, and *B*, (ii) compute the robust correlation between all pairs of these three variables using RobustCor function of the MRPC library with β=0.005 suggested by the authors; and (iii) call the MRPC function in the library with the above data table and robust correlation matrix, and different FDR cutoffs (ranging from 0 to 1, with a step size of 0.05) as its arguments. For each specified FDR cutoff, this function returns the edges among the three nodes of the triplet supported by the data and controlled at this FDR level. Using these edges called at different FDR cutoffs across all the triplets in a benchmark, we can then compute the PR curve of MRPC and associated AUPRC.

For the yeast benchmark, we also tried an alternative batch-wise running of MRPC, wherein MRPC was applied separately to each batch of benchmark triplets sharing the same *A* gene. In detail, for each distinct *A* gene in the benchmark, we selected all triplets involving this *A* gene; organized data on the SNP *L* corresponding to the *A* gene, the *A* gene itself, and all *B* genes in the selected triplets into a data table; computed all pairwise robust correlations among the variables in the table to obtain a correlation matrix; and finally called the MRPC function with arguments: the above data table and correlation matrix, and different FDR cutoffs (ranging from 0 to 1 in steps of 0.05 as in triplet-wise running). The resulting edges among the genes called at different FDR levels were aggregated across all batches to compute the PR curve—the associated AUPRC was poorer than when MRPC was run triplet-wise on the yeast benchmark, and hence we didn’t pursue batch-wise running further.

### 2.6. Application of NLCD to human data: Processing steps

To investigate the potential of NLCD to discover *in vivo* human causal gene networks, we applied NLCD to the GTEx (version 8) human multi-tissue genomic dataset. This dataset comprises transcriptomic measurements from multiple tissue samples obtained postmortem from several hundred human donors, and matched genetic data from the same human individuals. In our applications of NLCD, this human dataset posed challenges due to heterogeneity inherent to a real-world population (i.e., individual-to-individual differences in genetic ancestry, environmental exposures, demographic factors, etc.) and multiple testing burden across the tested triplets. Mitigating these challenges required the selection of tissues with large sample sizes (to tackle reduction of statistical power due to heterogeneity), careful selection of triplets (prior to NLCD’s application to reduce multiple testing burden), and increasing the resolution of p-values (to allow small p-values to survive multiple testing correction), as explained next.

**Selection of tissues**: We selected 10 tissues with the largest sample sizes, and applied NLCD to each of these tissues separately. These top 10 tissues (along with their sample sizes shown within parentheses) are: Skeletal muscle (706), Whole blood (670), Sun-exposed skin (lower leg) (605), Tibial artery (584), Subcutaneous adipose (581), Thyroid (574), Tibial nerve (532), Not sun-exposed skin (suprapubic) (517), Lung (515), and Esophagus mucosa (497). Note that these sample sizes are based on the transcriptomic data available in the GTEx Analysis V8 portal’s open access section named “Single-Tissue *cis*-eQTL Data”, which we refer to as the GTEx data section hereafter.

**Selection of triplets (for a given tissue)**: For any given tissue, we assemble the list of triplets (*L*,*A*,*B*) to be tested as follows. We first download from the GTEx data section, the list of *cis*-eQTL relations detected in the tissue at FDR 5% (i.e., q-value cutoff of 0.05 as recommended in the portal). Using the resulting set of *cis*-eQTL SNPs, we next find all significant *trans*-eQTL relations involving any of these SNPs. From these results, we assemble the tissue-specific list of triplets to be tested, by pairing each *cis*-eQTL gene *A* with each *trans*-eQTL gene *B* that are both linked to the same SNP *L*. A *trans*-eQTL relation (*L*,*B*) is considered significant if its association p-value < 10^-5^ and the gene *B* is located at least 1 million base pairs away from *L*. *Trans*-eQTL relations are detected using the R package Matrix eQTL [37], which requires input data on genotype, gene expression, covariates, gene location, and SNP location. Genotype data was extracted from VCF (Variant Call Format) files using VCFTools, following GTEx data-access approval. Other input data such as expression data and covariate data (including genotype principal components and PEER factors) were downloaded from the GTEx data section. Triplets whose eQTL SNP *L* has three distinct genotype values are treated as diploid; those with two distinct values are treated as haploid; and those with one distinct value are excluded from testing.

**Filtering/Making causal calls (for a given tissue)**: For any given tissue, for each selected triplet explained above, we apply NLCD in both directions, L→A→B and L→B→A, to obtain two p-values. Here and in the steps below, *A* refers to the *cis*-eQTL gene and *B* the *trans*-eQTL gene linked to *L*. We explain how these two p-values are used to make causal calls, after certain filters are applied to reduce multiple testing burden and increase the reliability of causal calls.

- We first improved the resolution of NLCD’s four component tests’ p-values and thereby the final p-value using EPEPT as explained in Section 2.2.6 Optional postprocessing step, in order to allow small p-values to remain significant after correction for multiple testing.
- After applying EPEPT, to reduce multiple testing burden and increase confidence in our causal calls, we restricted further analysis to only triplets satisfying three filtering criteria: (i) a Test 3 p-value ≤0.1, since testing for causality is unnecessary when *A* and *B* are not correlated; (ii) at least 10 samples for each genotype value; and (iii) high mappability for both genes in the triplet (≥0.75 each) and low cross-mappability (0) between them, as sequence similarity between genes can lead to false-positive causal calls [38]. We also tried a relaxed version of the third criteria by additionally including triplets with unknown or unavailable (NA) values for mappability or cross-mappability in the database [39] used to obtain these measures.
- Consider the triplets retained after the above filtering—their L→A→B test p-values were corrected for multiple testing using the Benjamini-Hochberg method, whereas the reverse L→B→A test p-values were left uncorrected, in order to make more stringent causal calls. Specifically, we call a retained triplet as causal (L→A→B) if its corrected L→A→B p-value is ≤0.1 and its (uncorrected) L→B→A p-value is > 0.1. Recall that *A* is a *cis*- and *B* a *trans*-eQTL gene linked to *L*; our workflow therefore focuses on making causal calls in the *cis* → *trans* direction, though we acknowledge that *trans*-to- *cis* causal relationships may also exist biologically.

## 3. Results

### 3.1. Our NLCD method overview

NLCD determines if there is a causal relationship between two variables *A* and *B* (e.g., gene expression or other traits) using a third instrumental variable *L* (e.g., a genetic factor correlated to both the traits) by conducting four statistical tests and taking the maximum of these tests’ p-values as the final p-value (see Methods and Fig 1). Our NLCD’s main contribution is accounting for nonlinear causal relationship between the trait variables in these tests using nonlinear regression (unlike earlier linear causal discovery methods) and employing a novel conditional feature importance score in the fourth test for conditional independence. NLCD can work with any nonlinear regression model, and we have focused on KRR, SVR and ANN in this study (see Methods). The final call on whether data from an input triplet *L*, *A*, *B* supports a causal, reactive, or independent model is done based on NLCD’s final p-value as explained in Methods (see 2.2.5 Making the final call).

### 3.2. NLCD outperforms SOTA methods in simulated nonlinear benchmarks

We evaluated our NLCD method’s variants and other SOTA methods on their ability to distinguish causal vs. independent triplets in the simulated benchmark dataset (for sample size *n* = 500 in main text, and other sample sizes in supplement; see also Methods section 2.5.1 Classification task and benchmark datasets). Different types of simulated benchmark datasets correspond to different types of relationship between *A* and *B*, i.e., either a linear relation or a nonlinear relation that takes the form of a sine or sawtooth curve (see Methods section 2.3 Generation of simulated datasets and Fig 2A).

Among the three variants of our NLCD method applied on different simulated benchmark datasets, we see from Fig 2B that NLCD using KRR for nonlinear regression gives a better AUPRC than SVR and ANN. We used 500 permutations in these evaluations, and hence we will be using NLCD (KRR) with 500 permutations for further analysis, and refer to it simply as NLCD when it is clear from the context. We also observed NLCD’s performance across different hyperparameter configurations. In this sweep, NLCD (KRR)’s default hyperparameter configuration of (regularization strength α=1, RBF kernel with bandwidth γ=1) used throughout the manuscript, achieved performance comparable to the best-performing configurations, thereby indicating that the reported results are robust (see Section 2.2 in S1 Text NLCD performance across hyperparameter configurations, and Fig K in S1 Text).

**Fig 3:**
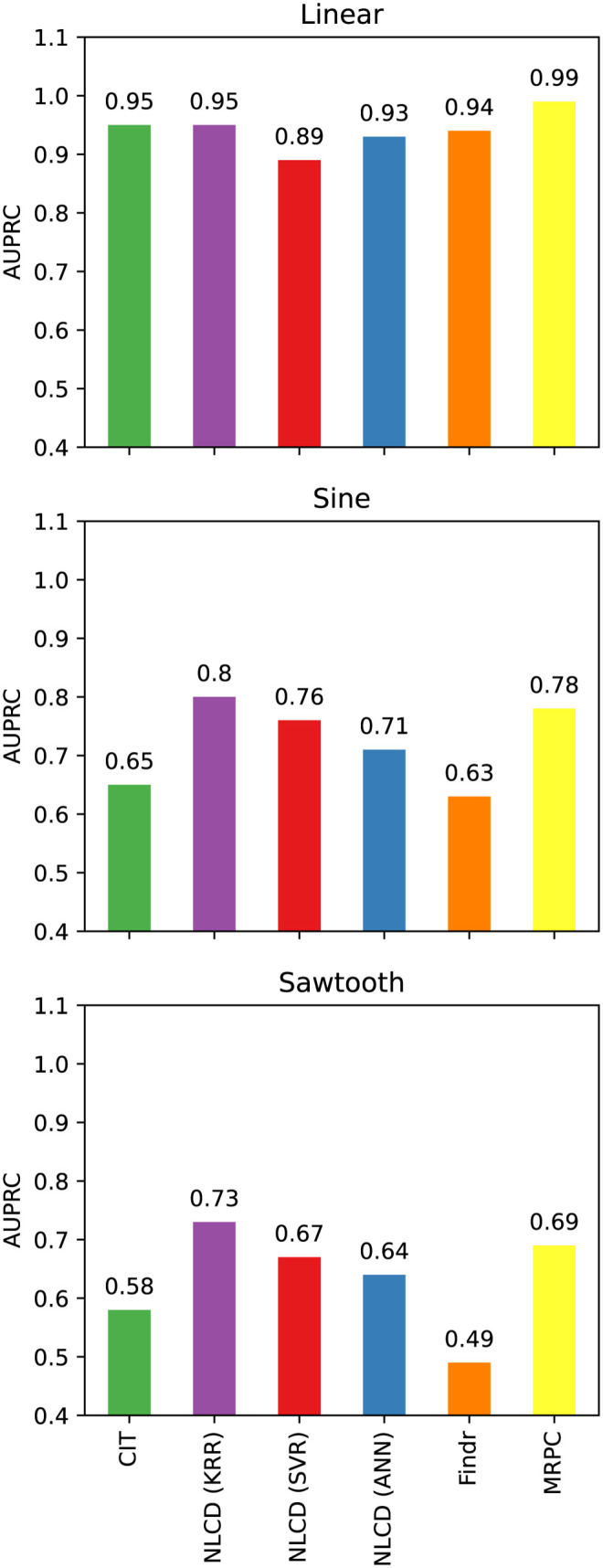
Comparison of NLCD with SOTA methods on simulated benchmark datasets. Simulated benchmarks comprising data on independent triplets vs. causal triplets (with linear, sine, or sawtooth causal relationship) were used to determine the classification performance (AUPRC) of different causal discovery methods (see also Fig A(a) in S1 Text for the underlying PR curves). NLCD and CIT were run using 500 permutations (see also Fig A(b) in S1 Text for results using 100 permutations). Sample size of *n* = 500 is used here.

We next compared NLCD variants with other SOTA methods for classifying triplets in the simulated benchmark dataset as causal vs. independent, and report the resulting AUPRC and Precision-Recall plots in Figs 3 and A(a) in S1 Text respectively. We see that NLCD fares well in all the cases, both linear and nonlinear. MRPC does marginally better than NLCD in linear and slightly worse than NLCD in nonlinear. We observe that all the methods perform comparably in the linear benchmarks, and CIT and Findr are worse than other methods in the nonlinear benchmarks. These results were obtained with the number of permutations set at 500 for CIT and NLCD; changing the number of permutations to 100 for both methods didn’t affect the relative performance of CIT and NLCD (see Fig A(b) in S1 Text). As discussed already, the results presented in the main text are on simulated benchmark datasets of sample size *n* = 500. See Table B in S1 Text for the AUPRC values of all methods for other sample sizes too.

### 3.3. Detailed evaluation of NLCD relative to the CIT baseline

To complement the comparative evaluations of our NLCD against multiple SOTA methods, we also performed more comprehensive comparison of NLCD against CIT, the baseline method that was extended to develop NLCD, using simulated benchmark datasets.

**Robustness of performance trends in simulated benchmarks**: We first checked whether the better performance of NLCD over CIT on simulated nonlinear (sine or sawtooth) benchmarks persists despite the stochastic nature of both methods. As NLCD and CIT rely on permutation testing, their empirical p-values can change if they are run with different random seeds on the same dataset. To evaluate if the performance trends observed above for one run remain similar across runs, we ran NLCD and the CIT for 10 different runs on the same dataset. The resulting PR curves (see Fig B(a) in S1 Text) show that the performance trends indeed persist despite the methods’ run-to-run variations.

We also checked variability in performance due to variations in the dataset. Ten datasets were simulated using the same generative model (see Methods) but independently using different random seeds. We can see from Fig B(b) in S1 Text that the PR curves of both NLCD and CIT across these 10 datasets show appreciable variability, still NLCD performs better than CIT on nonlinear (sine and sawtooth) benchmarks. The above observations on run-to-run variation and dataset variation hold in the case of both 100 and 500 permutations, and demonstrate that the performance improvement of NLCD method over the CIT baseline on nonlinear benchmarks is robust.

**Comparing the individual tests of NLCD vs. CIT**: To further understand how NLCD extends CIT, we compared the individual tests of these methods. We specifically compared the third test of NLCD and CIT (see Fig C in S1 Text), which checks for association between *A* and *B* for each value of *L*. It is observed that across linear triplets, CIT’s and NLCD’s p-values are well correlated; however, across nonlinear (sine or sawtooth) triplets, the p-values of CIT are spread across the y-axis (0–1) whereas that of NLCD remain very close to zero. Hence, NLCD works as designed to detect a nonlinear association between *A* and *B* better than CIT. The two methods can also differ in their fourth test, a key test of conditional independence, as follows. For many nonlinear causal triplets, CIT finds that *L* provides additional information on top of *A* to predict *B*; but this is not the case and *A* alone is sufficient to predict *B* if nonlinearity is properly modeled, which is what NLCD is designed to do and also achieves it via a low CFI score (see also a parabola triplet example below).

**Performance on unequal variance simulated datasets:** Since real-world causal triplets (e.g., yeast genomic data) can exhibit unequal conditional variances of *A* given *L*, and also linear or parabola-like gene-gene relationships, we also tested NLCD and CIT on unequal variance linear/parabola simulated benchmarks. An unequal variance simulated benchmark dataset comprises the simulated dataset on 100 unequal variance linear (or parabola) triplets (see Methods) and another on the 100 equal variance independent triplets. PR curves on this unequal variance benchmark (shown in Fig E(a) in S1 Text for sample size *n* = 500) of NLCD is better than that of CIT, since the former is explicitly designed to handle variance heterogeneity across genotype values. In contrast, both methods perform similarly on equal variance linear and parabola simulated benchmarks (*n* = 500; see Methods and Fig E(a) in S1 Text). Although parabolic relations are nonlinear, they more closely resemble linear associations than sine or sawtooth relations; the linear baseline CIT hence performs comparably to NLCD on the equal variance parabola benchmark. All of these performance trends hold for other sample sizes of 300 and 1000 as well (see Fig F in S1 Text).

To illustrate how NLCD handles unequal variances, we simulate an example parabola triplet with unequal conditional trait variances, and plot the predictions of *B* made by NLCD and CIT using *A* and *L* as inputs (see Fig E(b), E(c), and E(d) in S1 Text). In the overlap region of these plots, NLCD’s predictions are almost identical between *L* = 0 vs. *L* = 1, whereas CIT’s predictions depend on the value of *L*. Hence for NLCD, *L* doesn’t supplement *A* to predict *B* and thereby results in a low CFI score as mentioned above; whereas for CIT, its linear modeling of a truly nonlinear *A*-*B* relationship results in *L* providing information on top of *A* to predict *B*.

### 3.4. NLCD distinguishes the direction of causality

Benchmark evaluations were done so far in only one direction, i.e., from A→B. To check if NLCD can infer the direction of causality, simulated data on a benchmark triplet was fed to NLCD separately in the causal direction (L→A→B) and also in the anti-causal direction (L→B→A). Fig D in S1 Text shows that NLCD can distinguish between these directions for causal (linear, sine and sawtooth) triplets, since final p-values output by NLCD are mostly near zero in the causal direction, but spread across the range from 0 to 1 or closer to 1 in the anti-causal direction. For independent triplets which is not causal in either direction, NLCD’s p-values in both directions were near one and thereby supported neither a causal nor reactive model. These observations show that NLCD can correctly identify the causal direction.

### 3.5 NLCD’s performance on the yeast benchmark and its nonlinear subsets

Given the promising performance of our method on simulated benchmark data, we next tested it using real-world yeast genomic data, collected from 1012 yeast segregants. On yeast benchmark datasets comprising causal and independent triplets (see Section 2.4 Yeast benchmarking methodology), we tested whether NLCD and SOTA methods can correctly classify an input triplet as causal vs. independent using genomic data on the triplet. First, note that on the full yeast benchmark dataset, comprising both linear and nonlinear gene-gene associations, Findr and CIT had the highest performance (in terms of AUPRC) followed closely by NLCD and then MRPC (see Fig G in S1 Text).

**Fig 4:**
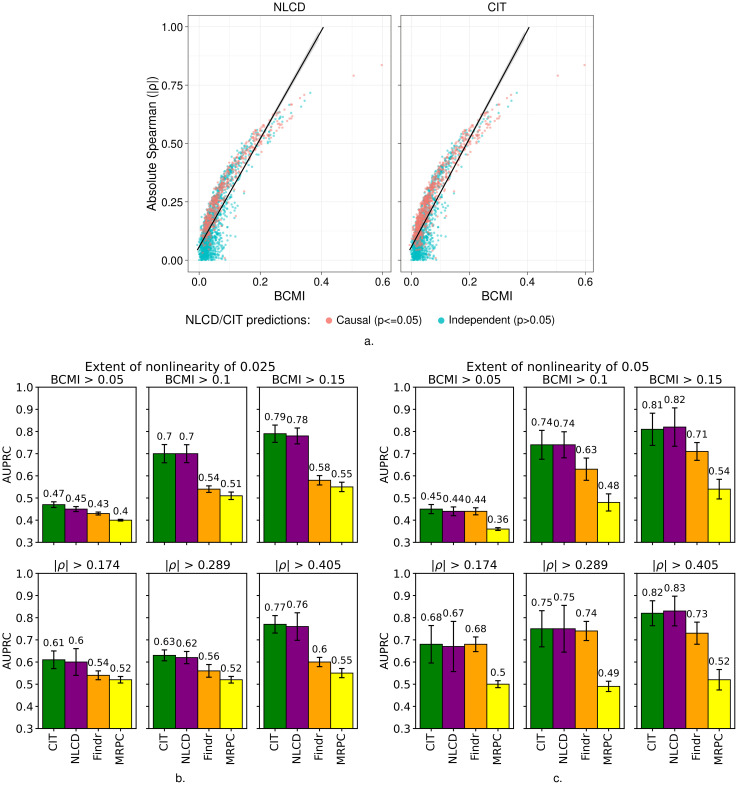
Comparison of NLCD with SOTA methods on the yeast benchmark and its subsets. **a.** For all causal triplets in the full yeast benchmark (i.e., known TF-TG causal pairs recorded in a ground-truth database), plotted here is the strength of linear (absolute Spearman) vs. nonlinear (BCMI) gene-gene correlation calculated using data from 1012 yeast segregants. Colors indicate triplets predicted as causal vs. independent by NLCD (left) vs. CIT (right) at a (final) p-value cutoff of 0.05. Black line shows the fitted linear regression model. Similar plots are shown for all independent triplets in the full yeast benchmark in Fig H in S1 Text. **b & c.** On different (nonlinear) subsets of the yeast benchmark dataset, performance of causal discovery methods are shown (reported AUPRC is average and standard deviation across 10 random versions of the benchmark subset; random classifier’s AUPRC is 0.5 in all subplots). Here, we show the plots for an extent of nonlinearity (δ) of at least 0.025 and 0.05 (see Fig I in S1 Text for other cutoff values).

Would the performance trend change if we subset the yeast benchmark to focus on triplets whose genes exhibit sufficient correlation and importantly a high extent of nonlinearity? To answer this question, we assembled benchmark subsets as explained in Methods (Section 2.4.2 Yeast triplet subsets with different correlation strengths/types) by considering the strengths of linear (|ρ|) vs. nonlinear (BCMI) type correlation between the genes of triplets (shown in Figs 4A and H in S1 Text for all causal and all independent triplets respectively in the yeast benchmark). Fig 4A shows that genes in many causal triplets (i.e., ground-truth causal TF-TG pairs) have low |ρ| and low BCMI likely due to noise in genomic data and other factors (see Methods); so it is sensible to filter the benchmark subsets to remove these poorly correlated triplets, which cannot be recovered reliably by any method (including NLCD and CIT as shown in the figure). Another important filter applied on the benchmark subsets retains only triplets with relatively stronger nonlinear than linear type correlation (i.e., more typical BCMI than |ρ| values) by applying a lower-bound cutoff on the extent of nonlinearity δ (e.g., δ>0.025; see Methods).

Across several subsets of the yeast benchmark dataset with different degrees of nonlinearity, our NLCD and the CIT method performed comparably/better than other methods (see Fig 4B and 4C, and similar figures for more δ cutoffs in Fig I in S1 Text). In detail, for benchmark subsets compiled using low BCMI cutoff (e.g., BCMI > 0.05), AUPRC of all the methods are mostly worse than the random AUPRC of 0.50 for reasons already explained above pertaining to insufficiently correlated causal gene pairs. However at higher cutoffs on BCMI and across different cutoffs on δ, AUPRC improves appreciably for all the methods, with CIT and NLCD in particular showing consistently better performance than Findr. MRPC’s performance was lower and may be attributed to how it was run on the triplets (however note that we tried different ways of running MRPC on the yeast benchmark and report the best-performing one here; see Methods). NLCD is comparable to but not strictly better than CIT for the δ cutoffs tested, and may perform better in more nonlinear benchmark subsets where the δ cutoff is increased beyond 0.05. But such subsets could not be assembled due to the relatively small number of nonlinear triplets in the yeast benchmark. In summary, taking all the results together, NLCD is a competitive method for recovering causal triplets from this real-world yeast genomic data.

### 3.6. Discovering causal gene relations across multiple human tissues

The promise of NLCD is to detect causal relations under *in vivo* human settings, where interventions are not possible and observational data is the main resort to discover causality. But heterogeneity in a human population that leads to weak instruments and multiple testing burden across all tested triplets poses challenges, and necessitates large sample sizes and careful selection of triplets. So, we applied NLCD to only the 10 human tissues with the most number of samples in GTEx and filtered the triplets using *cis*/ *trans*-eQTL associations (see Methods 2.6 Application of NLCD to human data: Processing steps). Furthermore, we increased resolution of the NLCD tests’ p-values by using 1000 instead of the default 500 permutations and postprocessing the resultant test statistics using the EPEPT method (see Methods 2.2.6 Optional postprocessing step).

**Fig 5:**
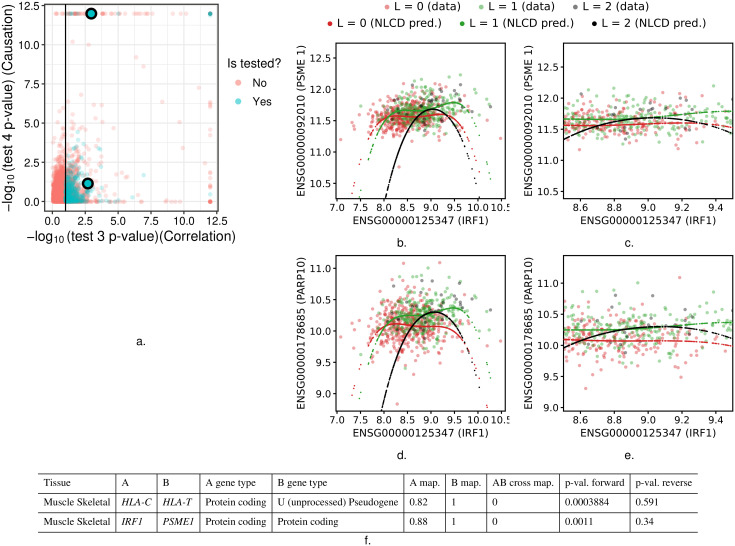
Application of NLCD to human muscle GTEx data. **a.** Strength of causal vs. correlative relationship of the gene pair in each muscle triplet, as measured via the corresponding NLCD tests’ p-values, is shown. Only triplets satisfying certain filtering criteria (see Methods 2.6 Application of NLCD to human data: Processing steps) were tested; and colored as indicated in the legend. The black line with x-intercept of 1 corresponds to a p-value cutoff of 0.1. The two black-circles indicate the gene pairs *IRF1*- *PSME1* and *IRF1*- *PARP10*. **b & c.** NLCD’s predictions (denoted pred) of *PSME1* using *IRF1* and different values of *L* as inputs is plotted (left), along with a zoomed view of this plot (right). **d & e.** NLCD’s predictions (denoted pred) of *PARP10* using *IRF1* and different values of *L* as inputs is plotted (left), along with a zoomed view of this plot (right). **f.** Listed are all muscle triplets detected as causal by NLCD based on stringent p-value (pval.) and mappability/cross-mappability (map./cross map.) thresholds. Dot colors denote the genotype dosage L∈0,1,2 (red, green, black respectively); the same colors are used for the data points and for NLCD’s predictions, as indicated in the legend at the top of panels b–e.

We first ran our NLCD workflow on the skeletal muscle tissue triplets, which had a sample size of 706, and summarize the results in Fig 5. Specifically, we plot in Fig 5A the p-values from test 3 (of conditional association) and test 4 (of conditional independence) for these muscle triplets, as these test 3 and 4 p-values are indicative of correlation and causation respectively. This plot shows that only a subset of the correlated gene pairs show strong evidence of causality, thereby emphasizing the importance and challenge of distinguishing causality from correlation in human datasets of moderate sample sizes. At stringent p-value and mappability/cross-mappability thresholds (see Methods), we discovered two causal triplets (Fig 5F). One of them is the *IRF1-PSME1* pair – it is known from literature [40] that *IRF*1 is a transcription factor regulating a target gene *PSME*1. When inspecting the data and NLCD predictions for this regulating pair (see Fig 5B and 5C), we see that the NLCD predictions for all three values of *L* are close to each other in the overlap region (where the IRF1 values corresponding to these three *L* values overlap), thereby leading to the critical conditional independence test 4 passing. To provide a counterexample, we show similar plots (Fig 5D and 5E) for a pair (*IRF1-PARP10*) that is not causal but correlated, to indicate how the NLCD predictions for different values of *L* are different in the overlap region.

We next ran NLCD on all the 10 selected GTEx tissues, after relaxing the mappability/cross-mappability criteria (See Methods section: 2.6 Application of NLCD to human data: Processing steps). Note that with the original stringent criteria, NLCD could not pick up causal triplets in any non-muscle tissue – this could be due to lower sample sizes in non-muscle compared to muscle tissue. The relaxed criteria resulted in 14 causal triplets (Table 1). The genes in these triplets do not pertain to TF-TG pairs, and so are less likely to be direct causal pairs. Instead, these causal triplets may reflect indirect (multi-step propagation of) causality between the genes. For instance, one of the causal calls involve protein-coding pairs: *AP4B1*→*METTL5* in whole blood, which is plausible since changes in protein sorting regulated by *AP4B1* could possibly propagate to changes in translational regulation via *METTL5*. This protein-coding causal gene pair, along with another pair *POLI*→*NTNG1* in esophagus-mucosa, have not been characterized before to our knowledge, and we therefore present them as biologically plausible candidate causal hypotheses warranting follow-up in independent datasets and experimental systems. Another set of identified causal triplets involve long intergenic non-coding RNAs: the edge *ZDHHC8P1*→*RP11-65J3.3*, in which *RP11-65J3.3* is a lincRNA appears in two skin tissues (sun-exposed and not-sun-exposed); a further lincRNA-containing edge, *AC024560.2*→*SDHAP2*, is found in sun-exposed skin. lincRNAs have a wide documented repertoire of cis- and trans-regulatory mechanisms [41], providing a plausible mechanistic basis for these calls. Finally, most of the discovered pairs involving pseudogenes cannot be dismissed as mappability-related artifacts, as the role of pseudogenes as genuine regulators has been suggested earlier [42]. Our predicted causal relations, both on pseudo genes and protein-coding or lincRNA genes, provide promising candidate hypotheses, rather than established regulatory edges, that could be tested further using other datasets and follow-up experiments.

**Table 1:** Human causal triplets detected by NLCD under relaxed criteria. Causal triplets detected by NLCD in the selected human GTEx tissues are listed as in Fig 5F, but after relaxing a filtering criterion: triplets with unknown (NA) mappability/cross-mappability values for their genes are not removed. All other filtering criteria and significance cutoffs remain the same as before (see Methods 2.6 Application of NLCD to human data: Processing steps). Abbreviations: Skin Sun Exposed Lower Leg (Skin SEL), Skin Not Sun Exposed Suprapubic (Skin SES), Esophagus-Mucosa (EM), Transcribed Unprocessed (TU), Unprocessed (U), lincRNA (long intergenic non-coding RNA).

Tissue	A	B	A gene type	B gene type	A map.	B map.	AB cross map.	p-val. forward	p-val. reverse
Muscle Skeletal	*HLA-C*	*HLA-T*	Protein coding	U Pseudogene	0.82	1	0	0.0003884	0.591
Whole Blood	*HLA-L*	*HLA-K*	TU Pseudogene	U Pseudogene	0.97	0.97	NA	6.029e-07	0.541
Whole Blood	*HLA-K*	*HLA-S*	U Pseudogene	U Pseudogene	0.97	NA	NA	3.319e-05	0.485
Whole Blood	*HLA-L*	*HLA-W*	TU Pseudogene	U Pseudogene	0.96	0.98	NA	3.213e-05	0.503
Whole Blood	*AP4B1*	*METTL5*	Protein coding	Protein coding	0.98	NA	NA	0.000698	0.861
Whole Blood	*HLA-K*	*XXbac-BPG248L24.12*	U Pseudogene	U Pseudogene	0.97	NA	NA	9.897e-05	0.174
Skin SEL	*ZDHHC8P1*	*RP11-65J3.3*	TU Pseudogene	lincRNA	NA	NA	NA	0.0001872	0.436
Skin SEL	*AC024560.2*	*SDHAP2*	lincRNA	TU Pseudogene	NA	NA	NA	7.138e-07	0.333
Skin SES	*FRG1CP*	*FAM182B*	U Pseudogene	Protein coding	NA	NA	NA	1.548e-05	0.273
Skin SES	*PDXDC2P*	*NPIPB10P*	TU Pseudogene	U Pseudogene	NA	NA	NA	4.535e-05	0.146
Skin SES	*ZDHHC8P1*	*RP11-65J3.3*	TU Pseudogene	lincRNA	NA	NA	NA	0.000782	0.836
Lung	*CH507-9B2.9*	*AP001056.1*	Protein coding	lincRNA	NA	NA	NA	6.577e-05	0.129
EM	*SDHAP2*	*AC024560.2*	TU Pseudogene	lincRNA	NA	NA	NA	2.895e-05	0.82
EM	*POLI*	*NTNG1*	Protein coding	Protein coding	NA	NA	NA	0.0006454	0.421

## 4. Conclusion and Discussion

We’ve developed a method NLCD to account for nonlinearity when discovering causal relationship between two gene expression or clinical traits. NLCD works under an MR-like framework wherein a genetic factor associated with the two tested traits is used to infer the presence of causality from observational data on this triplet. The evidence for causal relationship between the traits/genes in the triplet is summarized using a p-value based on four statistical tests.

Existing SOTA methods for the triplet-based causal discovery problem assume linear relationship between the genes. NLCD, by virtue of its NLR modeling and CFI scoring to realize the statistical tests, shows promising performance on nonlinear simulated and real-world benchmarks. Specifically, in simulated benchmark datasets comprising triplets with nonlinear gene-gene causal relationships, NLCD achieves better AUPRC than existing SOTA methods. For triplets with linearly related genes, NLCD performs comparably to other methods. In real-world yeast benchmarks, NLCD is competitive with other methods in recovering triplets with linear or nonlinear TF-TG causal relationships. Application of NLCD to every pair of genes with a common eQTL SNP provides a workflow to uncover the gene regulatory network underlying a biological system, and we illustrate this approach on human multi-tissue genomic data to detect tissue-specific causal genes.

Some caveats about NLCD’s performance are worth mentioning. NLCD is developed by extending a linear causal discovery method CIT. Whereas NLCD clearly outperformed the linear baseline CIT in simulated nonlinear benchmarks, NLCD and CIT performed similarly on real-world yeast triplets. This may be due to the predominantly linear causal relationship between TFs and TGs in the yeast genomic dataset. NLCD’s advantage over CIT is likely to become more pronounced in practice, if the real-world datasets involve complex clinical traits, unequal conditional trait variances, or time-series measurements of periodic genes. Like other triplet-based causal discovery methods, NLCD relies on the exclusion restriction that *L* affects *B* only through *A* (thereby satisfying the conditional independence between *A* and *B* given *L*); under horizontal pleiotropy, NLCD does not return a causal call unless the indirect L→A→B effect substantially dominates the direct L→B leakage.

Application to human GTEx data showed that NLCD is likely to be useful in practice for detecting reliable causal relations only when sample sizes of cohorts are sufficiently large (close to 1000s) to overcome weak genetic instruments and multiple testing burden, and stringent filters are applied to reduce false positives driven by (cross-)mappability issues. NLCD was able to overcome these issues to a reasonable extent and thereby detect reliable causal relations using yeast segregants’ and human GTEx data; larger sample sizes of observational data and longitudinal measurements in future studies such as biobank-scale human studies can make it easier for NLCD to discover causal networks.

We now make explicit the settings in which NLCD is expected to provide a clear advantage over linear alternatives, and the scope of its application beyond gene–gene triplets. NLCD is expected to outperform linear methods in three regimes. (i) *Strongly nonlinear or non-monotonic trait–trait relationships:* on the simulated sine and sawtooth causal benchmarks, as seen in the simulated data; such non-monotonic signals, e.g., from circadian-regulated or saturating dose response genes, are precisely where linear MR methods lose information that NLCD’s NLR modeling and CFI scoring retain. (ii) *Heteroscedasticity across genotype strata:* on the unequal-variance simulated benchmarks (Figs E and F in S1 Text), NLCD substantially improves over CIT on both linear- and parabola-unequal-variance triplets (iii) *Complex or biobank-scale clinical traits:* the comparable yeast performance of NLCD and CIT is consistent with the predominantly linear yeast TF–TG relationships, and we expect the gap to widen on richer phenotypes (quantitative clinical measurements, polygenic disease outcomes, longitudinal/time-series expression of periodic genes).

Beyond gene-level applications, NLCD is fundamentally a causality test between any two continuous traits *A* and *B* anchored by a valid instrument *L*; it makes no assumption that *A* and *B* are gene expression measurements. The same workflow therefore applies directly to protein–metabolite or gene–metabolite triplets (with *L* a genetic variant linking a protein/gene mediator to a metabolite outcome), epigenetic–expression triplets (with *A* a CpG methylation or chromatin-accessibility measurement and *B* a downstream gene expression level), classical MR settings for clinical exposure–outcome traits where nonlinear relationships have been repeatedly reported [10,13], and other multi-omics triplets (e.g., SNP–transcript–splicing) provided *L* is a valid instrument for both downstream variables.

We motivate certain future directions to improve NLCD methodology further. The running time of NLCD (KRR) increases cubically with sample size and linearly with the number of permutations (see Tables C and D in S1 Text). We could incorporate faster KRR methods [43] into NLCD as it could help us run more permutations in a given time period and thus obtain higher resolution p-values. NLCD’s current default is to use 500 permutations, the minimum number recommended in an earlier study [44]. Another future work pertaining to NLCD methodology would be to conduct its tests 3 and 4 (i.e., compute the respective test statistics of MSE loss and CFI score) using a testing dataset. Currently, the whole dataset on a triplet $D_{T}$ is used to train the model as well as calculate the test statistics. In the future, we could assess whether the already competitive performance of NLCD on benchmarks can be further improved by splitting $D_{T}$ into a training and a testing dataset for model fitting and test statistics computation respectively. A further methodological direction is to integrate covariate adjustment directly into NLCD. At present, NLCD operates on residualized data: covariates such as experimental batch and growth-rate for yeast (Section 2.4) and PEER factors and genotype principal components for human GTEx data (Section 2.6) are regressed out upstream using a linear model, following standard practice in the MR/causal-inference literature. Replacing this upstream linear adjustment with a nonlinear covariate adjustment performed jointly with NLCD’s NLR fitting could capture nonlinear confounding effects that the current pipeline misses, particularly in heterogeneous human cohorts spanning batches, tissues, sexes, or ages.

Theoretical studies on the sample complexity of NLCD could be another direction of future work. Specifically, given our generative model of data from a causal triplet, can we derive a lower bound on the number of samples *n* required to detect this causal triplet with high probability? This lower bound could be a function of the strength of the causal relation, the size of (i.e., the fraction of *n* datapoints that lie in) the overlap region between different values of *L*, and other relevant factors. Finally, while we have focused on CIT as the baseline to extend upon for nonlinear causal discovery, we can also develop a nonlinear extension of any other triplet-based causal discovery method by incorporating the core concepts of NLCD (viz., NLR modeling and CFI scoring) in their methodology.

NLCD makes a minimal set of assumptions and simple choices for the NLR models to determine the presence of causality. To assess whether NLCD is robust against deviations from its simple distributional assumptions, we inspected NLCD’s behavior on simulated datasets whose conditional distributions deviated from normality (see Section 2.3 in S1 Text NLCD’s tests under departures from normality, and Tables E and F in S1 Text). These results showed that NLCD can recover causal triplets even when the genes do not follow a normal distribution. However, we note that NLCD’s reliance on permutation testing implies that the different steps of NLCD are modular and therefore its distributional assumptions can be changed in a fairly straightforward manner if required. NLCD is also model-agnostic with respect to NLR, and nonlinearity can be modeled using any other suitable model besides the three used here.

To conclude, we have developed a method NLCD for observational-data-driven linear and nonlinear causal discovery, and demonstrated its competitiveness in diverse datasets. These results hold promise for its future applications to rapidly accumulating data on genetic/molecular/clinical variables from large (e.g., biobank-scale) studies to reveal causal biological networks operating *in vivo*.

## Supporting information

S1 TextSupplementary Methods, Results, Tables, and Figures.This single supporting file contains all supplementary material referenced in the main text: the Supplementary Methods and Results sections (Sections 1.1–2.3), Tables A–F and Figs A–K in S1 Text. Legends for each component are listed below.List of legends for Supplementary Methods: **Section 1.1 Background on underlying theorem and model. Section 1.2 Specifications of the nonlinear models used. Section 1.3 Calculation of probabilities for CFI score. Section 1.4 Overlap region of triplet gene distributions conditioned on genotype.** List of legends for Supplementary Results: **Section 2.1 Assessing NLCD’s control of Type I error. Section 2.2 NLCD performance across hyperparameter configurations. Section 2.3 NLCD’s tests under departures from normality.**List of legends for Supplementary Tables: **Table A. Example dataset of a triplet:** For a triplet (*L*, *A*, *B*), this table shows an example dataset with each row being an observation of the triplet. Each observation comprises values of the genotype variable *L* and trait variables *A* and *B* in a sampled individual. **Table B. Performance of causal discovery methods on simulated benchmark datasets across different sample sizes.** AUPRC values of methods, NLCD, CIT, Findr, and MRPC, for classifying the triplets in different simulated benchmark datasets of varying sample sizes as causal vs. independent. Shown in bold are the best performing method’s AUPRC for each sample size and type of relation (linear, sine and sawtooth) of the causal triplets in the benchmark. **Table C. Running time of NLCD:** Running time taken by NLCD for different number of permutations and sample sizes. NLCD was run on Supermicro SSG-6039P-E1CR16H server with 112 cores, each containing an Intel(R) Xeon(R) Platinum 8180 2.50GHz CPU; and a total RAM of 1TB. Parallelization of triplets’ processing was achieved by using the multiprocessing library of Python. **Table D. Runtime complexity of NLCD:** Time complexity of the four statistical tests in NLCD in terms of *n*, the number of samples, and *M*, the number of permutations. **Table E. NLCD results stratified by the normality of *A:*** Number of triplets split according to the non-normality of *A* gene and the causal calls made by NLCD. Lilliefors test p-value of less than 0.05 suggests that the distribution is not normal. These are the same (equal variance) datasets underlying the simulated benchmarks studied in the main text, with sample size *n* = 500; and NLCD was run using 100 permutations. **Table F. NLCD results stratified by the normality of *B:*** Number of triplets split according to the non-normality of *B* gene and the causal calls made by NLCD. Lilliefors test p-value of less than 0.05 suggests that the distribution is not normal. These are the same (equal variance) datasets underlying the simulated benchmarks studied in the main text, with sample size *n* = 500; and NLCD was run using 100 permutations.List of legends for Supplementary Figures: **Fig A. PR curves of causal discovery methods applied on simulated benchmark datasets.** a. PR curve of different methods for sample sizes 300, 500, and 1000, with the number of permutations for CIT and NLCD set at 500. b. PR curve of NLCD and CIT, in particular, with the number of permutations set at 100. Note that the *y*-axis range of panel b is different from that in panel a to zoom into the plots. **Fig B. Performance of CIT/NLCD under run-to-run and dataset-to-dataset variations.** a. For each type of simulated benchmark, a single benchmark dataset is simulated, and the PR curves of 10 runs of NLCD and of CIT on this single dataset are shown. b. For each type of simulated benchmark, 10 benchmark datasets are simulated (using different random seeds), and the PR curves of a single run of NLCD and of CIT on these 10 datasets are shown. All simulated benchmark datasets used here have sample size *n* = 500, and NLCD and CIT were run using either 100 or 500 permutations as indicated in the figure. **Fig C. Comparison of “Test 3” p-values of CIT/NLCD:** The hexagonal bin plots compare p-values of the conditional association test (Test 3) of CIT and its corresponding test in NLCD across the causal triplets of different simulated benchmark datasets (with sample size *n* = 500). **Fig D. Bidirectional application of NLCD:** Comparison of p-values of NLCD when applied in A→B or B→A direction on simulated datasets that are generated in the A→B direction. These are the same datasets underlying the simulated benchmarks studied in the main text, with sample size *n* = 500. **Fig E. Effect of unequal variance on CIT vs. NLCD, and closer inspection of a parabola causal triplet.** a. Shown are the PR curves of CIT and NLCD run using 100 permutations on linear and parabola simulated benchmarks with sample size *n* = 500, and conditional variances of *A* being unequal (first two plots) or equal (next two plots) given different values of *L*. b. Plot of an example triplet, specifically a simulated unequal variance causal parabola triplet. c. NLCD’s prediction of *B* from *A* and *L* for the unequal variance parabola triplet in panel b. d. CIT’s prediction of *B* from *A* and *L* for the unequal variance parabola triplet in panel b. In panels c and d, the term “pred” stands for prediction, and red dots indicate the *B* predictions using *L* = 0 and the corresponding *A* values, and green dots the *B* predictions using *L* = 1 and the corresponding *A*. **Fig F. Effect of unequal variance on CIT and NLCD under different sample sizes.** Performance of CIT and NLCD on linear and parabola simulated benchmark datasets, when the variances of the *A* gene conditioned on different genotype (*L*) values are equal (bottom) vs. unequal (top). The benchmark datasets had a sample size of 300 or 1000 as indicated within parentheses in the legend; and the methods were run using 100 permutations. **Fig G. PR curves on the full yeast benchmark:** PR curves of NLCD and SOTA methods on the task of classifying triplets in the full yeast benchmark dataset as causal vs. independent. The full benchmark includes triplets with linear and/or nonlinear type gene-gene correlations. The curves are plotted using hyperbolic interpolation, and the corresponding AUPRC values are: 0.55 (Findr), 0.55 (CIT), 0.53 (NLCD), and 0.51 (MRPC). Random classifier’s AUPRC is 0.50. **Fig H. NLCD and CIT predictions on the yeast benchmark’s independent triplets and all triplets.** For all independent triplets in the full yeast benchmark (i.e., TF-TG pairs recorded as independent/non-causal in a ground-truth database; see Methods in main text), plotted here is the strength of linear (absolute Spearman) vs. nonlinear (BCMI) gene-gene correlation calculated using data from 1012 yeast segregants. Colors indicate triplets predicted as causal vs. independent by NLCD (left) vs. CIT (right) at a (final) p-value cutoff of 0.05. Black line shows the fitted linear regression model. **Fig I. Comparison of NLCD with SOTA methods on further (nonlinear) subsets of the yeast benchmark.** On different (nonlinear) subsets of the yeast benchmark dataset, performance of causal discovery methods are shown (reported AUPRC is average and standard deviation across 10 random versions of the benchmark subset; random classifier’s AUPRC is 0.5 in all subplots). Here, we show the plots for an extent of nonlinearity (δ) of at least 0, 0.0125, and 0.0375, to complement similar plots shown in the main text for other δ cutoffs. **Fig J. Empirical assessment of NLCD’s control of Type I error:** This figure assesses the calibration of NLCD component tests’ p-values and the combined $p_{final}$ on a simulated dataset of 100 independent triplets (with each triplet’s sample size being *n* = 1000). Specifically, for each of NLCD’s four component tests (a–d) and for the combined test (e), the histogram of p-values across 100 independent simulated triplets is shown. The dashed gray line marks the per-bin count expected under a Uniform[0,1] distribution (10 per bin). Each panel title reports the mean p-value; and frac<0.05, the fraction of triplets whose p-value is below 0.05. **Fig K. NLCD (KRR)’s AUPRC across 45 hyperparameter configurations:** For the 45 hyperparameter configurations of KRR considered in the NLCD (KRR) method, this figure shows the AUPRC for distinguishing nonlinear (sine) triplets from independent triplets in the simulated benchmark dataset. The configurations swept over kernel function/type, regularization strength α, kernel parameter γ, and polynomial degree. Sample size of *n* = 500, and 500 permutations were used. **Top-left:** linear kernel (γ and degree not applicable), with AUPRC reported as a function of α; **Top-middle and top-right:** RBF and Laplacian kernels, with AUPRC displayed as a heatmap over the α×γ grid; the red outline on the RBF panel denotes the default α=1, γ=1 configuration used throughout the manuscript. **Bottom-left and bottom-middle:** polynomial kernel, with γ∈{0.1,1.0}, with AUPRC reported over the α×degree grid. All heatmaps share a common viridis color scale (**bottom-right**), and cell values denote AUPRC.(PDF)

## References

[1] Pearl J. Causality: Models, Reasoning, and Inference. 2nd ed. Cambridge University Press; 2009.

[2] Klungel OH, Martens EP, Psaty BM, Grobbee DE, Sullivan SD, Stricker BHC, et al. Methods to assess intended effects of drug treatment in observational studies are reviewed. J Clin Epidemiol. 2004;57(12):1223–31. doi: 10.1016/j.jclinepi.2004.03.011 15617947

[3] Millstein J, Zhang B, Zhu J, Schadt EE. Disentangling molecular relationships with a causal inference test. BMC Genet. 2009;10:23. doi: 10.1186/1471-2156-10-23 19473544 PMC3224661

[4] Schadt EE, Lamb J, Yang X, Zhu J, Edwards S, Guhathakurta D, et al. An integrative genomics approach to infer causal associations between gene expression and disease. Nat Genet. 2005;37(7):710–7. doi: 10.1038/ng1589 15965475 PMC2841396

[5] Wang L, Michoel T. Efficient and accurate causal inference with hidden confounders from genome-transcriptome variation data. PLoS Comput Biol. 2017;13(8):e1005703. doi: 10.1371/journal.pcbi.1005703 28821014 PMC5576763

[6] Chen LS, Emmert-Streib F, Storey JD. Harnessing naturally randomized transcription to infer regulatory relationships among genes. Genome Biol. 2007;8(10):R219. doi: 10.1186/gb-2007-8-10-r219 17931418 PMC2246293

[7] Badsha MB, Fu AQ. Learning causal biological networks with the principle of Mendelian randomization. Front Genet. 2019;10:460. doi: 10.3389/fgene.2019.00460 31164902 PMC6536645

[8] Badsha MB, Martin EA, Fu AQ. MRPC: An R Package for Inference of Causal Graphs. Front Genet. 2021;12:651812. doi: 10.3389/fgene.2021.651812 33995486 PMC8120292

[9] Kvamme J, Badsha MB, Martin EA, Wu J, Wang X, Fu AQ. Causal network inference of cis- and trans-gene regulation of expression quantitative trait loci across human tissues. Genetics. 2025;230(2):iyaf064. doi: 10.1093/genetics/iyaf064 40179003 PMC12135172

[10] Sulc J, Sjaarda J, Kutalik Z. Polynomial Mendelian randomization reveals non-linear causal effects for obesity-related traits. HGG Adv. 2022;3(3):100124. doi: 10.1016/j.xhgg.2022.100124 35832928 PMC9272036

[11] Sukumaran S, Almon RR, DuBois DC, Jusko WJ. Circadian rhythms in gene expression: relationship to physiology, disease, drug disposition and drug action. Adv Drug Delivery Rev. 2010;62(9-10):904–17. 10.1016/j.addr.2010.05.009 PMC2922481 20542067

[12] Burgess S, Davies NM, Thompson SG, EPIC-InterAct Consortium. Instrumental variable analysis with a nonlinear exposure-outcome relationship. Epidemiology. 2014;25(6):877–85. doi: 10.1097/EDE.0000000000000161 25166881 PMC4222800

[13] Arvanitis M, Qi G, Bhatt DL, Post WS, Chatterjee N, Battle A, et al. Linear and nonlinear mendelian randomization analyses of the association between diastolic blood pressure and cardiovascular events. Circulation. 2021;143(9):895–906. doi: 10.1161/circulationaha.120.049819 33249881 PMC7920937

[14] Zheng X, Aragam B, Ravikumar P, Xing EP. DAGs with NO TEARS: Continuous optimization for structure learning. Adv Neural Inform Process Syst. 2018;31.

[15] Zheng X, Dan C, Aragam B, Ravikumar P, Xing EP. Learning sparse nonparametric DAGs. In Proceedings of the 23rd International Conference on Artificial Intelligence and Statistics (AISTATS), volume 108 of Proceedings of Machine Learning Research. 2020. pp. 3414–25.

[16] Lachapelle S, Brouillard P, Deleu T, Lacoste-Julien S. Gradient-based neural DAG learning. In International Conference on Learning Representations (ICLR). 2020.

[17] Yu Y, Chen J, Gao T, Yu M. DAG-GNN: DAG structure learning with graph neural networks. In: Proceedings of the 36th International Conference on Machine Learning (ICML), volume 97 of Proceedings of Machine Learning Research. 2019.

[18] Zhang K, Peters J, Janzing D, Schölkopf B. Kernel-based conditional independence test and application in causal discovery. In Proceedings of the Twenty-Seventh Conference on Uncertainty in Artificial Intelligence (UAI). AUAI Press; 2011. pp. 804–13.

[19] Hoyer PO, Janzing D, Mooij JM, Peters J, Schölkopf B. Nonlinear causal discovery with additive noise models. Adv Neural Inform Process Syst. 2008;21:689–96.

[20] Zhang K, Hyvärinen A. On the identifiability of the post-nonlinear causal model. In: Proceedings of the Twenty-Fifth Conference on Uncertainty in Artificial Intelligence (UAI). 2009.

[21] Li C, Shen X, Pan W. Nonlinear causal discovery with confounders. J Am Stat Assoc. 2024;119(546):1205–14. doi: 10.1080/01621459.2023.2179490 39077372 PMC11286219

[22] Gao M, Ding Y, Aragam B. A polynomial-time algorithm for learning nonparametric causal graphs. In: Advances in Neural Information Processing Systems. Vol 33. 2020. pp. 11599–611.

[23] Koller D, Friedman N. Probabilistic Graphical Models: Principles and Techniques. The MIT Press; 2009.

[24] Pedregosa F, Varoquaux G, Gramfort A, Michel V, Thirion B, Grisel O, et al. Scikit-learn: Machine learning in Python. J Mach Learn Res. 2011;12:2825–30.

[25] Watson DS, Wright MN. Testing conditional independence in supervised learning algorithms. Mach Learn. 2021;110(8):2107–29. doi: 10.1007/s10994-021-06030-6

[26] Casella G, Berger RL. Statistical Inference. Duxbury Press; 2002.

[27] Knijnenburg TA, Lin J, Rovira H, Boyle J, Shmulevich I. EPEPT: a web service for enhanced P-value estimation in permutation tests. BMC Bioinformatics. 2011;12:411. doi: 10.1186/1471-2105-12-411 22024252 PMC3277916

[28] Knijnenburg TA, Wessels LFA, Reinders MJT, Shmulevich I. Fewer permutations, more accurate P-values. Bioinformatics. 2009;25(12):i161-8. doi: 10.1093/bioinformatics/btp211 19477983 PMC2687965

[29] Albert FW, Bloom JS, Siegel J, Day L, Kruglyak L. Genetics of trans-regulatory variation in gene expression. Elife. 2018;7:e35471. doi: 10.7554/eLife.35471 30014850 PMC6072440

[30] Ludl A-A, Michoel T. Comparison between instrumental variable and mediation-based methods for reconstructing causal gene networks in yeast. Mol Omics. 2021;17(2):241–51. doi: 10.1039/d0mo00140f 33438713

[31] Teixeira MC, Viana R, Palma M, Oliveira J, Galocha M, Mota MN, et al. YEASTRACT+: a portal for the exploitation of global transcription regulation and metabolic model data in yeast biotechnology and pathogenesis. Nucleic Acids Res. 2023;51(D1):D785–91. doi: 10.1093/nar/gkac1041 36350610 PMC9825512

[32] Pardy C. Mutual information as an exploratory measure for genomic data with discrete and continuous variables. The University of New South Wales; 2013.

[33] Pardy C, Galbraith S, Wilson SR. Integrative exploration of large high-dimensional datasets. Ann Appl Stat. 2018;12(1). doi: 10.1214/17-aoas1055

[34] Davis J, Goadrich M. The relationship between Precision-Recall and ROC curves. In: Proceedings of the 23rd International Conference on Machine Learning, ICML 2006. New York, NY, USA: Association for Computing Machinery; 2006. pp. 233–40.

[35] Flach PA, Kull M. Precision-Recall-Gain curves: PR analysis done right. In Proceedings of the 29th International Conference on Neural Information Processing Systems - Volume 1, NIPS 2015. Cambridge, MA, USA: MIT Press; 2015. pp. 838–46,

[36] Wang L, Michoel T. Whole-Transcriptome Causal Network Inference with Genomic and Transcriptomic Data. In: Sanguinetti G, Huynh-Thu VA, editors, Gene Regulatory Networks: Methods in Molecular Biology. New York, NY: Springer New York; 2018. pp. 95–109. 10.1007/978-1-4939-8882-2_4 30547397

[37] Shabalin AA. Matrix eQTL: ultra fast eQTL analysis via large matrix operations. Bioinformatics. 2012;28(10):1353–8. doi: 10.1093/bioinformatics/bts163 22492648 PMC3348564

[38] Saha A, Battle A. False positives in trans-eQTL and co-expression analyses arising from RNA-sequencing alignment errors. F1000Res. 2018;7:1860. doi: 10.12688/f1000research.17145.2 30613398 PMC6305209

[39] Saha A, Battle A. Mappability resources for GRCh38 using Gencode v26 annotation. figshare. Dataset. 2018. 10.6084/m9.figshare.7315136.v3

[40] Shi L, Perin JC, Leipzig J, Zhang Z, Sullivan KE. Genome-wide analysis of interferon regulatory factor I binding in primary human monocytes. Gene. 2011;487(1):21–8. doi: 10.1016/j.gene.2011.07.004 21803131 PMC3167955

[41] Ulitsky I, Bartel DP. lincRNAs: genomics, evolution, and mechanisms. Cell. 2013;154(1):26–46. doi: 10.1016/j.cell.2013.06.020 23827673 PMC3924787

[42] Pink RC, Wicks K, Caley DP, Punch EK, Jacobs L, Carter DRF. Pseudogenes: pseudo-functional or key regulators in health and disease? RNA. 2011;17(5):792–8. doi: 10.1261/rna.2658311 21398401 PMC3078729

[43] Alaoui AE, Mahoney MW. Fast randomized kernel ridge regression with statistical guarantees. In: *Proceedings of the 29th International Conference on Neural Information Processing Systems - Volume 1*, NIPS 2015. Cambridge, MA, USA: MIT Press; 2015. pp. 775–83.

[44] Marozzi M. Some remarks about the number of permutations one should consider to perform a permutation test. *Statistica*. 2024;64(1):193–201.

