# Supplementary material for: NLCD: A method to discover nonlinear causal relations among genes
Source: PLoS Comput Biol. 2026 Jul 31;22(7):e1014570. doi: 10.1371/journal.pcbi.1014570 (PMC13450862; doi:10.1371/journal.pcbi.1014570)
Supplement: Supplementary file 2 [file pcbi.1014570.s002.pdf]

Dear Reviewers, Academic Editor Yuehua Cui, and Section Editor Pedro Mendes,

We thank the reviewers for their careful evaluation and constructive comments on our work, and the editors for securing valuable reviews of our work and granting us the opportunity to revise the manuscript. We have addressed all reviewer comments through new analyses and revisions of the manuscript, and a summary of the key revisions is provided below.

- A new Introduction paragraph situates our proposed method (NLCD, NonLinear Causal Discovery) against the broader machine-learning causal-discovery literature.
- New robustness analyses of NLCD have been performed: hyperparameter sweep of NLCD's KRR (kernel ridge regression) version, a per-test p-value calibration check, a horizontal-pleiotropy simulation, sensitivity analysis of GTEx (Genotype-Tissue Expression) results across permutation counts and nonlinear regression model choices, and comparative analysis of per-component-test AUPRC (Area Under Precision-Recall Curve) of NLCD vs. CIT on yeast data.
- Results section now includes a paragraph on the biological plausibility of the GTEx discoveries, and the Discussion section explicitly describes the regimes in which NLCD outperforms linear alternatives and its applicability beyond gene–gene triplets.

Our point-by-point responses are provided below (in blue font); corresponding revisions are highlighted in yellow in the Marked-Up manuscript. Page numbers in our responses below refer to the revised manuscript, unless stated otherwise.

We have addressed all reviewer comments and believe that the resulting revisions have significantly strengthened the manuscript. We hope that the reviewers and editors share this view, and we look forward to your response.

Thank you,
Manikandan Narayanan
(on behalf of all co-authors)

****

Point-by-point response:

****

Reviewer #1:

I have a few comments and suggestions that I think would help clarify the contribution and improve the overall presentation of the manuscript:

1. The idea of extending CIT to handle nonlinear relationships is very interesting and well motivated. It would help to more clearly explain how NLCD differs from other nonlinear or ML-based causal discovery methods, and what it adds beyond existing approaches.

Response:

We thank the reviewer for raising this important question about positioning. We have added a new paragraph in the Introduction (highlighted in yellow in the revised manuscript) that situates NLCD against the broader ML (machine learning) literature on discovering causal DAG (directed acyclic graph) structures from observational data. This new paragraph is paraphrased below, with the actual paragraph in the revised manuscript in Page 2 citing all the necessary references and expanding the abbreviations.

In this new paragraph, we contrast NLCD with (i) score-based continuous optimization DAG learners (NOTEARS, GraN-DAG, DAG-GNN) that assume causal sufficiency (no hidden confounders assumption, which is often violated in real-world triplet-based genomic data), (ii) constraint-based methods such as PC with kernel-based conditional independence tests that cannot orient a confounded bivariate edge, (iii) bivariate functional causal models (ANM, PNL) that explicitly forbid hidden confounders, and (iv) methods that provide provable computational/statistical guarantees on recovering nonlinear DAGs but under certain restrictive assumptions --- DeFuSE algorithm learns a nonlinear DAG under the assumption of linear (Gaussian) hidden confounders and an additional sublinear growth condition on the functional causal relationships, whereas the NPVAR method of Gao et al. learns a nonparametric (including nonlinear) DAG but under the restrictive assumptions of equal-residual-variances and causal sufficiency (no unmeasured confounding). The paragraph closes by clarifying NLCD's positioning: rather than ruling out hidden confounding by assumption or adjusting it away under parametric constraints as in the methods mentioned above, NLCD uses the genetic instrument to test the conditional-independence implications of an unconfounded causal model, so that confounded triplets are rejected rather than oriented. This view can be further understood by referring to the Causal Equivalence Theorem in Supplementary Section 1.1 Background on underlying theorem and model).

2. The use of KRR, SVR, and neural networks is reasonable, and KRR appears to perform best in the benchmarks. Please explain why KRR was chosen as the default, and how sensitive results are to tuning or kernel choice.

Response:

We thank the reviewer for raising this point. KRR was chosen as the default because the simulation results show that it performs better than SVR and ANN.

To address whether NLCD's classification performance depends on the specific KRR hyperparameters, we performed a hyperparameter sweep over the following kernel types in KRR: linear, RBF (Radial Basis Function), polynomial, and Laplacian. A scan over the values of other hyperparameters besides the kernel type are as follows:

- Three different values of the regularization strength α were tried out for the linear kernel $\alpha = \{10^{-2}, 1, 10^2\}$.
- For the RBF and Laplacian kernels, α and the kernel parameter γ were varied as: $\alpha = \{10^{-2}, 1, 10^2\}$ and $\gamma = \{0.1, 1, 10, 100\}$.
- For the polynomial kernel, α, γ, and degree were varied as: $\alpha=\{10^{-2},1,10^2\}$, $\gamma =\{0.1, 1\}$, and degree=\{2, 3, 4\}.

This hyperparameter sweep showed that NLCD (KRR)'s performance is robust, i.e., not dependent on a finely tuned hyperparameter configuration. Findings from this analysis are described in brief in the main text in Page 10, and in more detail in Supplementary Results Section 2.2 and associated Fig. S11. Excerpt of the supplementary results section and Fig. S11 is shown below for the reviewer's convenience.

"*To assess the robustness of the reported NLCD (KRR) to the choice of hyperparameters of KRR, we performed a sweep over kernel type, regularization strength (α), kernel parameter (γ), and polynomial degree. Across 45 hyperparameter configurations, we computed the AUPRC for distinguishing nonlinear (sine) triplets from independent triplets in the simulated benchmark dataset. The default configuration of (α = 1, RBF kernel with γ = 1) used throughout the manuscript achieved performance comparable to the best-performing configurations, indicating that the reported results are not driven by a narrowly optimized choice of hyperparameters (see Fig. S11).*"

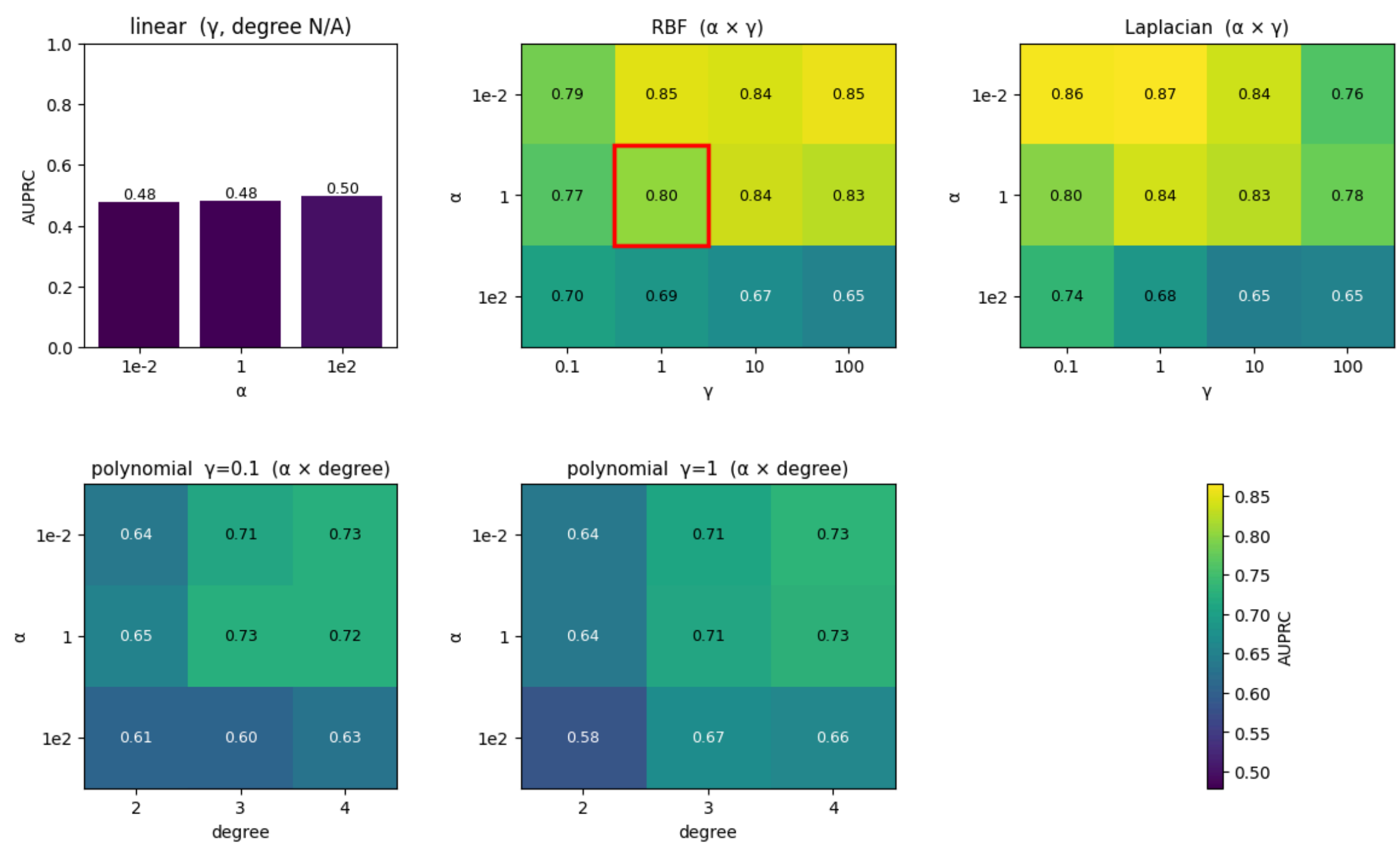

Fig. S11. NLCD (KRR)'s AUPRC across 45 hyperparameter configurations. For the 45 hyperparameter configurations of KRR considered in NLCD (KRR) method, this figure shows the AUPRC for distinguishing nonlinear (sine) triplets from independent triplets in the simulated benchmark dataset. The configurations swept over kernel function/type, regularization strength α,

kernel parameter γ, and polynomial degree. Sample size of n = 500, and 500 permutations were used. Top-left: linear kernel (γ and degree not applicable), with AUPRC reported as a function of α; Top-middle and top-right: RBF and Laplacian kernels, with AUPRC displayed as a heatmap over the α × γ grid; the red outline on the RBF panel denotes the default α = 1, γ = 1 configuration used throughout the manuscript. Bottom-left and bottom-middle: polynomial kernel, with γ ∈ {0.1, 1.0}, with AUPRC reported over the α × degree grid. All heatmaps share a common viridis color scale (bottom-right), and cell values denote AUPRC.

3. For me it would be helpful to include a clearer check of p-value calibration or type I error control, particularly for nonlinear but non-causal settings.

Response:

We agree that checking the calibration of p-values would be helpful. We've performed this check now and included the results in Supplementary Results Section 2.1. The excerpt of this supplementary results section, and the associated Fig. S10 is shown below for convenience.

"*We empirically assessed how NLCD's component permutation tests behave under the null hypothesis by applying NLCD on the simulated dataset of 100 independent triplets and inspecting the resulting p-values. The two unconditional association tests (Tests 1 and 2) have true alternatives because L is associated with both A and B in independent triplets. So these two tests' p-values are shifted toward zero. The two conditional independence tests (Tests 3 and 4) have true nulls: Test 4 p-values are approximately uniform, indicating nominal Type I error control; whereas Test 3 is mildly anti-conservative. The combined p-value from the intersection-union test (IUT), pfinal = max(p1, . . . , p4), is conservative. For instance, only one out of the 100 independent triplets is wrongly called as causal at cutoff* $p_{final} < 0.05$*, compared to the expected 5 triplets (see Fig. S10).*"

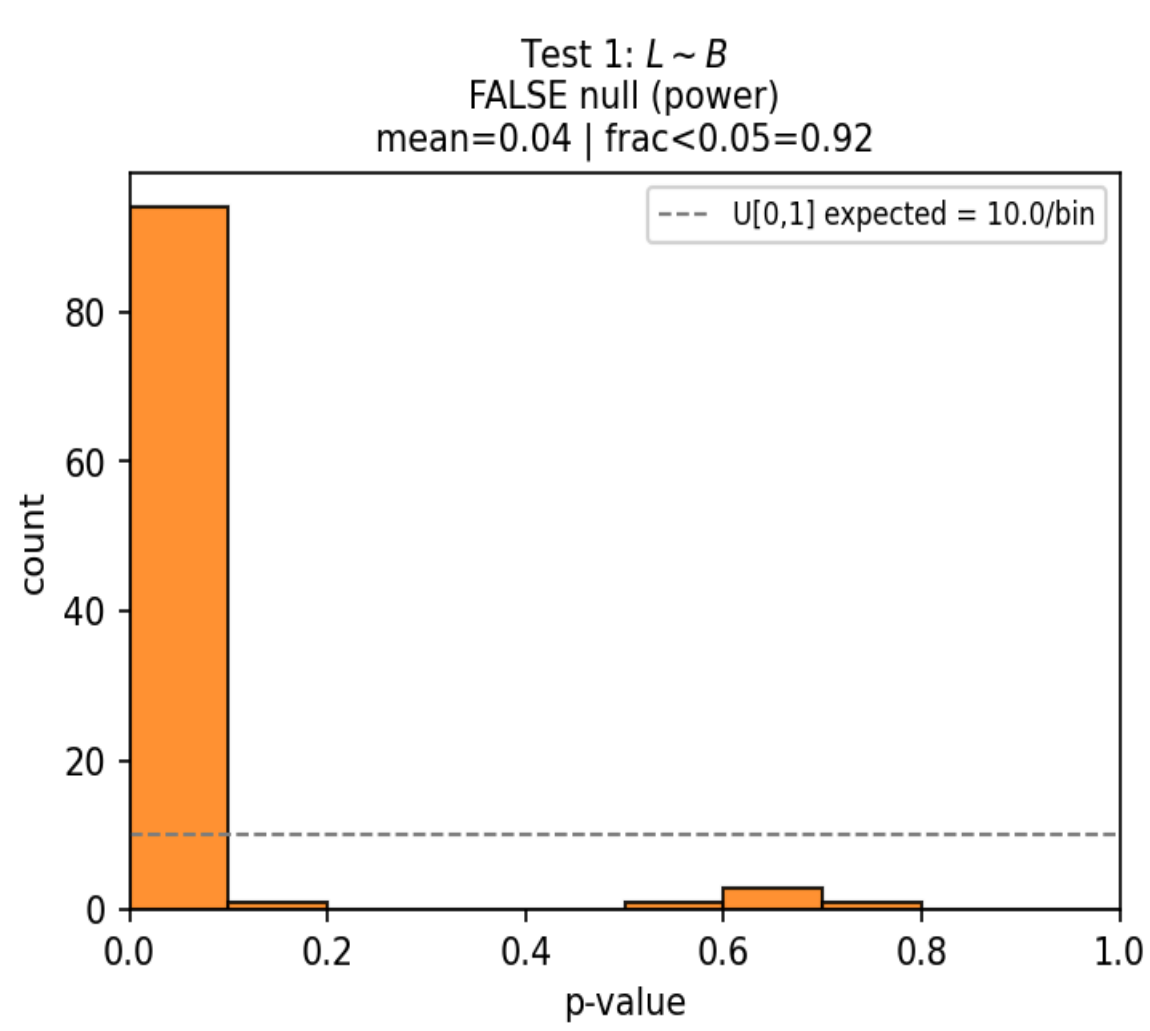

Test 1: L ~ B (alternative true)

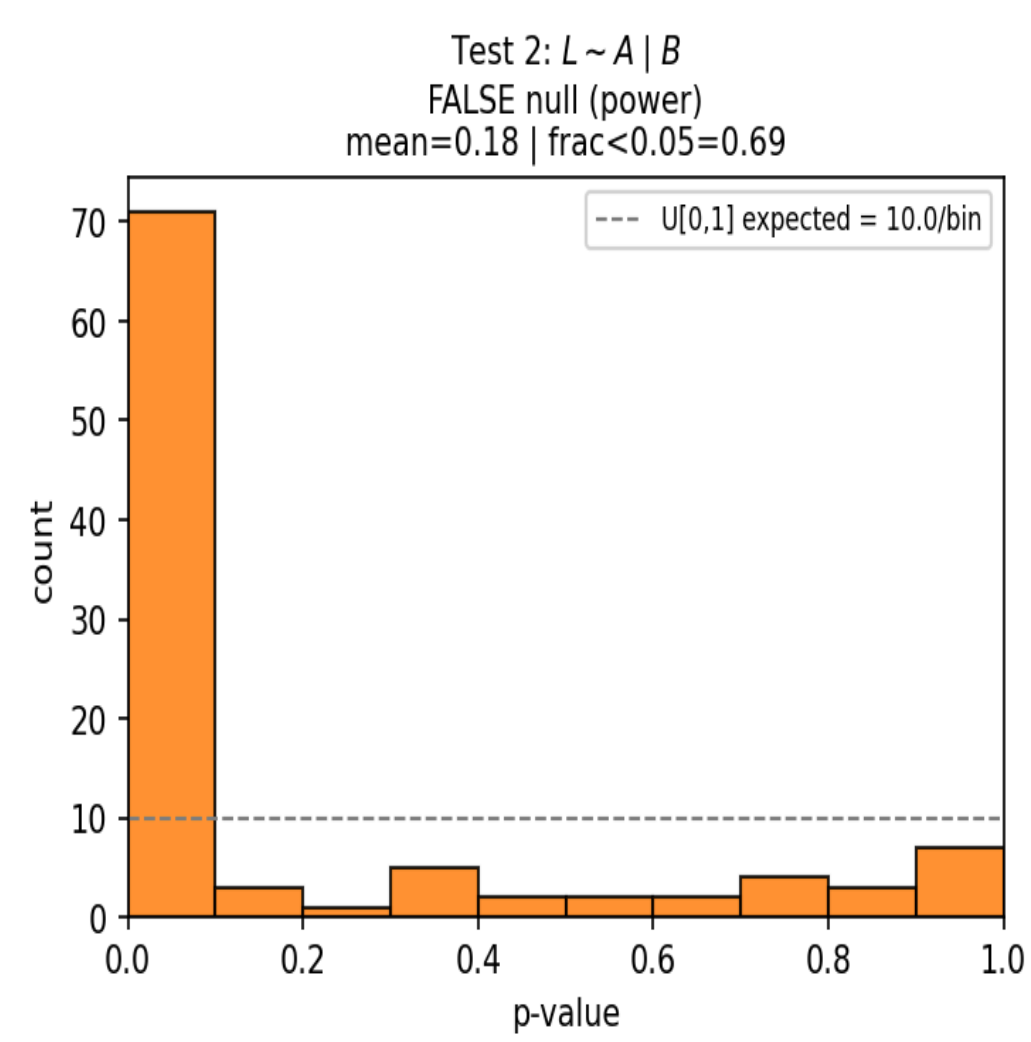

Test 2: L ~ A | B (alternative true)

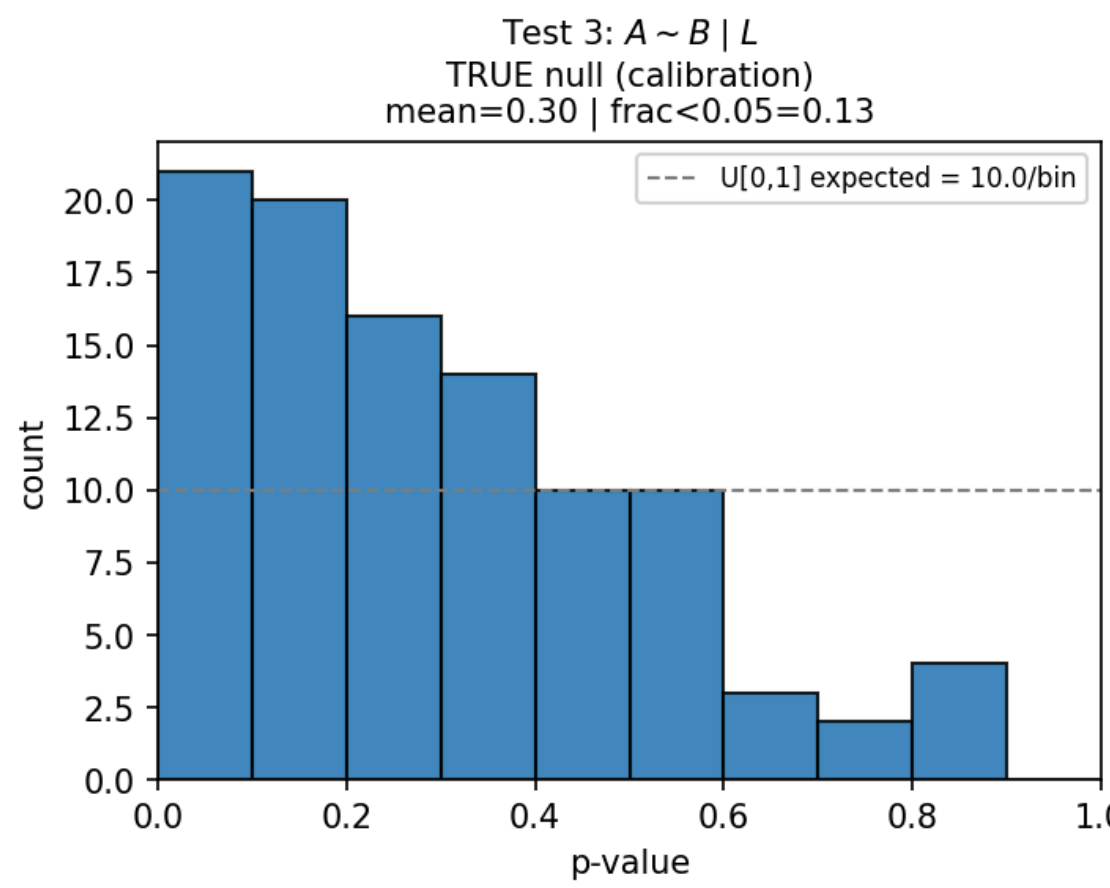

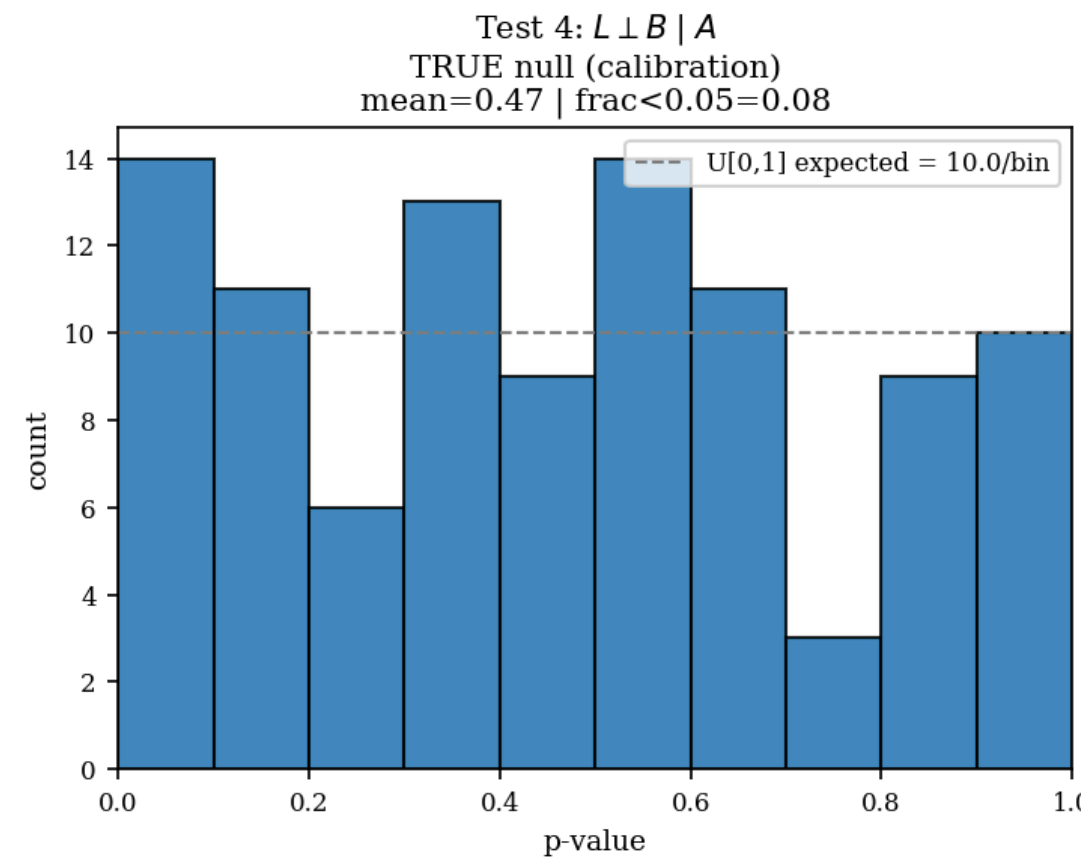

Test 3: A ~ B | L (null true)

Test 4: L ⊥ B | A (null true)

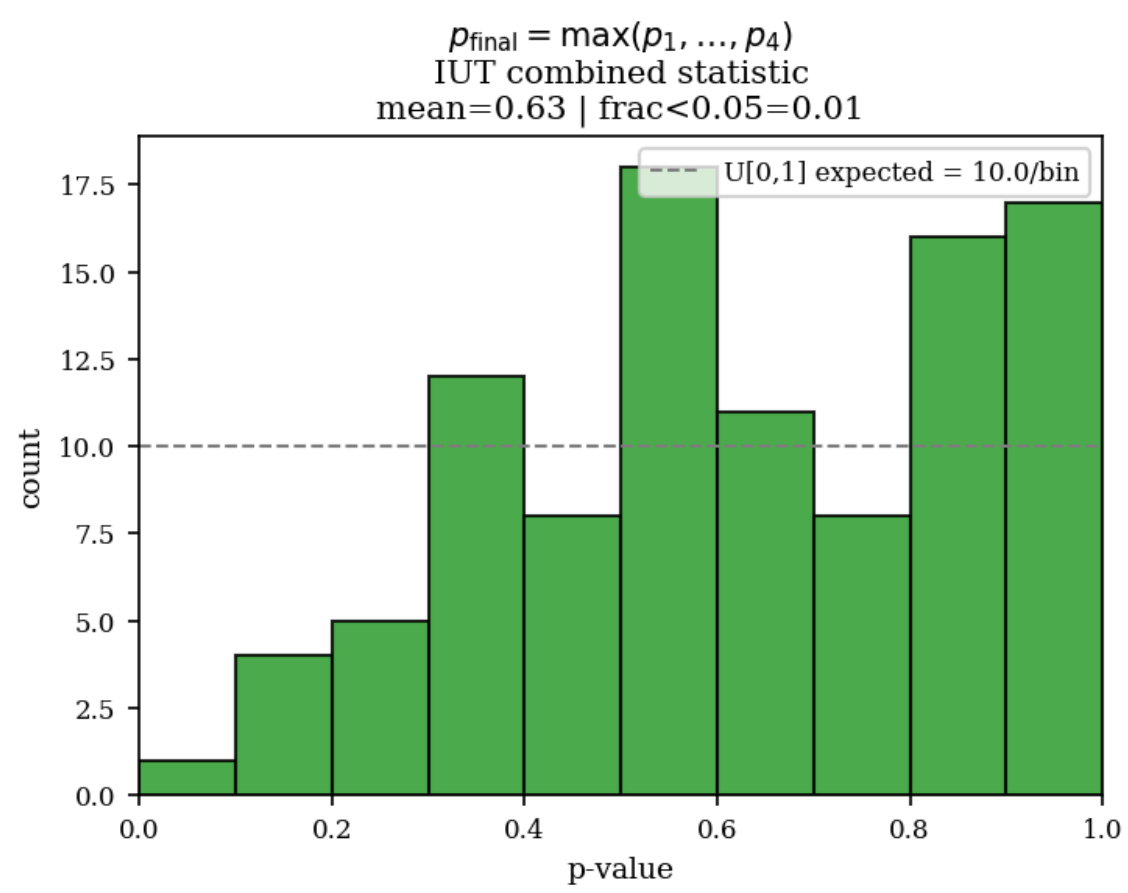

$p_{final} = \max(p1, \ldots, p4)$

Fig. S10. Empirical assessment of NLCD's control of Type I error. This figure assesses the calibration of NLCD component tests' p-values and the combined p_{final} on a simulated dataset of 100 independent triplets (with each triplet's sample size being n = 1000). Specifically, for each of NLCD's four component tests (a–d) and for the combined test (e), the histogram of p-values across 100 independent simulated triplets is shown. The dashed gray line marks the per-bin count expected under a Uniform[0,1] distribution (10 per bin). Each panel title reports the mean p-value; and frac < 0.05 the fraction of triplets whose p-value is below 0.05.

4. Conditional feature importance is a key part of the method. A bit more intuition about when CFI works well, and when it might struggle (for example, when there is little overlap between genotype groups), would improve clarity. Also since the proposed method involves nonlinear regression and many permutations, some discussion of computation time can help readers understand when NLCD is practical to use.

Response:

We thank the reviewer for this practical suggestion. We have addressed both points below and made minor corresponding edits to the manuscript in Page 4.

When CFI works well vs. when it may struggle. CFI is most informative when the overlap region between the conditional distributions (A | L = ℓ) across genotype strata is well populated: intuitively, CFI asks 'in the range of A where multiple genotype values are observed, does L still provide information about B once A is known?'. When genotype-group overlap is low - for example a strong eQTL effect that almost fully separates A across L values - few datapoints contribute to estimation of the conditional signal CFI, which leads to reduced power. We have added a few clarifying sentences to Section 2.2.3 describing this limitation and pointing to the overlap score as a simple diagnostic that practitioners can inspect prior to interpreting a CFI-based result.

Computation time. The manuscript already reports wall-clock running times of NLCD under different NLR models and sample sizes in Table S3, and running time complexity in Table S4.

5. The GTEx application is interesting, but some assessment of robustness (e.g., sensitivity to permutations, model choice) would increase confidence in these findings. In addition, a bit more discussion of biological plausibility would strengthen the real-data section.

Response:

We thank the reviewer for raising the query about the robustness of findings from applying NLCD (KRR) on the GTEx data. For the 15 triplets identified as causal in the GTEx application (union of triplets reported in Figure 5 and Table 1), we performed 3 new runs of NLCD using: KRR with 1000 permutations (with a different random seed than the one used in the paper), KRR with 10,000 permutations, and SVR with 1000 permutations. When comparing the resulting p-values with the original p-values reported in the paper (based on NLCD (KRR, 1000 permutations)), we find that 10 of the 15 triplets are also recovered by at least 2 of the 3 new runs as shown below, thereby supporting the robustness of our findings.

Tissue	A	B	p_fwd_paper	p_rev_paper	KRR_1000_fwd	KRR_1000_rev	KRR_10000_fwd	KRR_10000_rev	SVR_1000_fwd	SVR_1000_rev	# of new runs supporting the reported call
muscle	HLA-C	HLA-T	3.9E-04	5.9E-01	3.0E-03	5.8E-01	1.7E-03	5.9E-01	5.2E-02	6.0E-03	2
muscle	IRF1	PSME1	1.1E-03	3.4E-01	1.0E-03	3.7E-01	1.7E-03	3.6E-01	1.0E-03	4.0E-03	2
Whole_Blood	HLA-L	HLA-K	6.0E-07	5.4E-01	1.0E-03	5.4E-01	1.0E-04	5.5E-01	1.0E-03	7.9E-01	3
Whole_Blood	HLA-K	HLA-S	3.3E-05	4.9E-01	1.0E-03	1.0E-03	2.0E-04	3.0E-03	1.0E-03	8.0E-03	0
Whole_Blood	HLA-L	HLA-W	3.2E-05	5.0E-01	1.0E-03	5.0E-01	4.0E-04	5.0E-01	1.0E-03	4.1E-01	3
Whole_Blood	AP4B1	METTL5	7.0E-04	8.6E-01	1.0E-03	8.8E-01	1.6E-03	8.8E-01	2.3E-01	9.3E-02	2

Whole_Blood	HLA-K	XXbac-BPG248L24.12	9.9E-05	1.7E-01	1.0E-03	4.8E-02	1.0E-04	4.9E-02	1.0E-03	1.3E-01	1
Skin_Sun_Exposed_Lower_leg	ZDHHC8P1	RP11-65J3.3	1.9E-04	4.4E-01	2.0E-03	4.2E-01	5.0E-04	4.2E-01	2.0E-03	5.3E-01	3
Skin_Sun_Exposed_Lower_leg	AC024560.2	SDHAP2	7.1E-07	3.3E-01	1.0E-03	3.2E-01	1.0E-04	3.1E-01	1.0E-03	1.0E-03	2
Skin_Not_Sun_Exposed_Suprapubic	FRG1CP	FAM182B	1.5E-05	2.7E-01	1.0E-03	3.9E-02	2.0E-04	3.4E-02	1.0E-03	1.0E-03	0
Skin_Not_Sun_Exposed_Suprapubic	PDXDC2P	NPIPB10P	4.5E-05	1.5E-01	1.0E-03	1.7E-01	2.0E-04	1.6E-01	1.0E-03	9.8E-02	2
Skin_Not_Sun_Exposed_Suprapubic	ZDHHC8P1	RP11-65J3.3	7.8E-04	8.4E-01	1.0E-03	8.2E-01	2.0E-04	8.2E-01	1.0E-03	6.9E-01	3
Lung	CH507-9B2.9	AP001056.1	6.6E-05	1.3E-01	1.0E-03	4.1E-02	3.0E-04	4.7E-02	4.0E-03	1.2E-01	1
Esophagus_Mucosa	SDHAP2	AC024560.2	2.9E-05	8.2E-01	1.0E-03	8.0E-03	1.0E-04	8.2E-03	1.0E-03	1.0E-03	0
Esophagus_Mucosa	POLI	NTNG1	6.5E-04	4.2E-01	2.0E-03	4.2E-01	1.9E-03	4.4E-01	2.2E-02	1.8E-01	3

Table R1: For the 15 triplets called by NLCD (KRR, 1000 permutations) reported in the main text of the manuscript, we perform three new runs and report their p_{final} in the forward (fwd) and reverse (rev) directions here. For the new runs, the column names containing p-values are identified by {method}_{permutations}_{direction}, where the method (algorithm of NLCD) is either KRR or SVR, permutations (number of permutations used by NLCD) is 1000 or 10,000, and direction (direction of the triplets provided as input to the tests) is either fwd or rev. The columns p_fwd_paper and p_rev_paper refer to the p_{final} output by NLCD and used in the main text of the manuscript/paper. To calculate the final column, we consider a new run as supporting a triplet's causal call if the corresponding forward $p_{final} <= 0.1$ and reverse $p_{final} > 0.1$.

To address your second point, we have expanded on the biological plausibility of certain causal gene pairs identified by NLCD in human GTEx tissues. Please find the relevant text in Page 14 of the revised manuscript.

****

Reviewer #2:

The authors propose a method called NLCD, which extends the classical CIT framework to the nonlinear setting, and demonstrate its application to both human and yeast data. The manuscript is well written, and most methodological components are clearly explained.

Response:

Thanks for the positive comment on our writing, including methodological description.

I have several questions and suggestions that may help further clarify and strengthen the work.

1. How does NLCD perform under a structure such as B ← L → A, and at the same time A → B? In an MR framework, this corresponds to an invalid IV scenario where the instrument directly affects the outcome conditional on the exposure (horizontal pleiotropy)

Response:

We thank the reviewer for raising this important limitation. Horizontal pleiotropy — where L has a direct effect on B not mediated by A, in addition to A → B — violates the exclusion restriction (specifically the conditional independence between L and B given A) that underpins CIT, NLCD, and related causal discovery methods. So these methods, including NLCD, are not expected to detect causal relations under such scenarios (especially when the horizontal pleiotropy is strong). We have added a note about NLCD's expected performance under horizontal pleiotropy to the Conclusion and Discussion section in Page 14.

To specifically answer the reviewer's question, we illustrate NLCD's behavior under a pleiotropic data-generating process below using a simulated triplet (sample size n = 500):

$$L \sim \text{Bernoulli}(0.5)$$

$$A = \beta_{LA} \cdot L \ + \ \varepsilon_A, \quad \varepsilon_A \sim \mathcal{N}(0, 1)$$

$$B = \beta_{LB} \cdot L \ + \ \beta_{AB}\, f(A) \ + \ \varepsilon_B, \quad \varepsilon_B \sim \mathcal{N}(0, 1)$$

with $\beta_{LA} = 1$ (a moderately strong L → A effect, comparable to a strong cis-eQTL) and $\beta_{LB} = 1$ (a non-trivial direct L → B effect, i.e., genuine horizontal pleiotropy of comparable magnitude to the indirect path). For the result reported below, we used a linear function f(A) = A for simplicity:

We vary the relative strength of pleiotropy by varying the direct causal effect of A on B as follows, and show the causality p-value output by NLCD when applied on the 3 different triplets' data:

$\beta_{AB} = 1$ p_{final}=0.884

$\beta_{AB} = 10$ p_{final}=0.560

$\beta_{AB} = 100$ p_{final}=0.002

As expected, we see that NLCD fails to detect the causal relation using data from the first two triplets. However, as the causal effect of A on B dominates the pleiotropy effect from L, as in the third triplet, NLCD gets better at detecting the causal relation.

2. Since NLCD is built upon the CIT framework proposed by Millstein et al. (2009), it is important to clearly explain the rationale for the four tests described in Section 2.2.4. In particular, the exact null and alternative hypotheses for each test should be explicitly stated. This would make the logical structure of the procedure more transparent and help readers understand how the joint decision rule corresponds to the overall causal hypothesis.

Response:

We thank the reviewer for pointing out the need to explain the rationale of the tests and the need to explicitly state the null and alternative hypotheses for each test. We have added the rationale of the four tests in Section 2.2.4 (Realizing the four statistical tests of causality) in Page 4. In the same section, the different tests are explained with the null and alternative hypotheses stated.

3. Regarding Test 1, why is a nonlinear model necessary in that step? For example, in eQTL analysis, a simple linear regression is often sufficient to test the association between a variant and expression.

Response:

We thank the reviewer for this question and would like to clarify a potential source of confusion: **Test 1 in NLCD does not use nonlinear regression**. As the reviewer has indicated, a simple linear regression would suffice for this test. In our implementation, Test 1 is a likelihood-based association test of L ~ B realized using the Gaussian Negative Log Likelihood (NLL) test statistic. No nonlinear regression model is fit in this Test 1.

4. Another concern relates to potential overfitting. If the same dataset is used for both training the nonlinear model and evaluating the test statistic, this may introduce optimism bias. Have the authors considered cross-validation or an independent test set to construct the test statistic?

Response:

We thank the reviewer for raising this point. We use simple models such as KRR that are less prone to overfitting. So, our paper uses the full dataset for both training the model and evaluating the test statistics. To address your concern, we performed a new analysis on a simulated benchmark dataset (comprising 100 causal triplets with sine as nonlinear relation between genes A and B, and 100 independent triplets), wherein we modified our tests to train the models on

training data and calculate the statistics on the remaining test data. We list the AUPRC for different train fractions below. We observe that the AUPRCs are around similar values across these fractions, thereby supporting our current methodology in the paper which uses the full dataset for training the model and computing the statistics.

**Train Fraction**	**Test Fraction**	**AUPRC**
1.0 (full dataset)	1.0 (full dataset)	0.7981
0.8	0.2	0.7135
0.7	0.3	0.7611
0.6	0.4	0.7414
0.5	0.5	0.7684

5. In the yeast data application, the results suggest that NLCD does not outperform the original CIT, and even in the subset results, it is comparable to the CIT. Does this indicate that NLCD did not perform well in detecting linear relationship? It would be informative to compare NLCD and CIT at the level of the four component tests, for example by identifying which specific tests fail or differ between the two methods.

Response:

We agree with the reviewer that NLCD and CIT perform similarly in the yeast data application, but this doesn't imply that NLCD is not performing well in detecting linear relationships. CIT is a strong baseline for discovering linear relationships, and the fact that NLCD performs comparable to CIT in the yeast data (as shown in Figure 4) implies that NLCD does perform well in detecting linear relationships. Please note that NLCD also performs well in detecting linear relations in the simulated benchmark dataset.

To answer your second question, we computed the AUPRC of NLCD and CIT using p-values of each component test when applied on the full yeast benchmark dataset, which is dominated by linear relations as already mentioned in Page 14 in Conclusion and Discussion section. We see that the differences in the component test AUPRC between NLCD and CIT are very small — so NLCD and CIT perform comparably at the level of individual tests also when detecting predominantly linear relationships.

**Component test**	**AUPRC (CIT)**	**AUPRC (NLCD)**	**Δ (NLCD − CIT)**
Test 1: L ~ B	0.542	0.514	-0.028 ± 0.003
Test 2: L ~ A | B	0.499	0.499	0.000 ± 0.001
Test 3: A ~ B | L	0.517	0.516	-0.002 ± 0.001
Test 4: L ⊥ B | A	0.532	0.516	-0.016 ± 0.001
Overall p_{final} (reported in the paper):	0.546	0.525	-0.022 ± 0.002

6. For human data application, how do other SOTA methods perform in this application?

Response:

We thank the reviewer for this question. In the human data application using tissue-specific GTEx data, we do not have tissue-specific ground truth information on causal and independent gene pairs for evaluating the different methods (using AUPRC or other performance metrics). Hence, we did not compare NLCD with other SOTA methods on the human data application.

7. In Figure 5b–c, the authors state that "the NLCD predictions for all three values of L are close to each other in the overlap region," whereas in Figure 5d–e they indicate that predictions differ across values of L in the overlap region. However, the y-axis scales are not the same across panels, which makes it difficult to visually assess these differences. Clarification would be helpful. In addition, please specify what the colors of the dots represent in these plots.

Response:

We thank the reviewer for these comments, all of which have been addressed now. Specifically, we have updated the plot with the same scale and added legends for the dots.

8. Since the real-data results are largely comparable to existing methods, it would be helpful to briefly clarify in what settings NLCD is expected to provide clear advantages, for example, under stronger nonlinear relationships or more complex traits, and whether its intended scope extends beyond gene-level applications.

Response:

We thank the reviewer for this helpful suggestion. We have added two new paragraphs to the Conclusion and Discussion section in Page 14 that explicitly describes the scenarios where NLCD would likely perform better than alternatives, and also the scope of NLCD beyond gene-level applications. Here are excerpts from these new paragraphs:

"We now make explicit the settings in which NLCD is expected to provide a clear advantage over linear alternatives, and the scope of its application beyond gene–gene triplets. NLCD is expected to outperform linear methods in three regimes. (i) Strongly nonlinear or non-monotonic trait–trait relationships: <...text snipped…>. (ii) Heteroscedasticity across genotype strata: <...text snipped…>, (iii) Complex or biobank-scale clinical traits: the comparable yeast performance of NLCD and CIT is consistent with the predominantly linear yeast TF–TG relationships, and we expect the gap to widen on richer phenotypes (quantitative clinical measurements, polygenic disease outcomes, longitudinal/time-series expression of periodic genes)."

"Beyond gene-level applications, NLCD <...text snipped…>. The same workflow therefore applies directly to protein–metabolite or gene–metabolite triplets (with L a genetic variant linking a protein/gene mediator to a metabolite outcome), epigenetic–expression triplets (with A a CpG

methylation or chromatin-accessibility measurement and B a downstream gene expression level), classical MR settings for clinical exposure–outcome traits where nonlinear relationships have been repeatedly reported (PolyMR; NonlinearMR2), and other multi-omics triplets (e.g., SNP–transcript–splicing) provided L is a valid instrument for both downstream variables."

****

Reviewer #3:

This work represents a non-trivial development of a CIT-type mediation analysis approach for non-linear settings, particularly involving gene regulatory networks. The approach is well thought-out, implemented, and the rationale is clearly explained in the manuscript. Simulations demonstrate that the approach (NLCD) out performs the CIT, Findr, and MRPC in simulated non-linear scenarios and performs competitively to the CIT in linear settings. Software is provided via GitHub. It's my opinion that this will be an important and useful tool for the scientific community for identifying causal relationships between molecular features in non-linear settings, previously a methodological gap in this field.

Response:

We thank the reviewer for these highly positive assessments of our work.

The manuscript is well written and organized.

I have one comment that the authors could address, and that involves heterogeneity or experimental design-related factors that might need to be considered during the analysis. For example, what if there are batches, multiple tissues, cell types, genders, etc. How can this approach accommodate adjustment covariates?

Response:

We thank the reviewer for the comment. Currently, our approach can take in covariate-adjusted data and check for causality. For example, for the yeast data, we adjusted the data for two covariates (experimental batch and a growth rate covariate) using a linear model following standard practice in the community, and provided the adjusted data as input to NLCD for causal discovery. In future, we could explore including covariate adjustment within the NLCD framework itself, so that nonlinear covariate adjustments could also be explored. We have added text on this to the Conclusion and Discussion section in Page 16.

Minor comment: Change section 2.2 title from "Our NLCD Method" to something more informative, e.g., "NLCD Algorithm"

Response:

We thank the reviewer for the comment. We have changed it to "NLCD Algorithm" in the manuscript.

****
