# Supplementary material for: NLCD: A method to discover nonlinear causal relations among genes
Source: PLoS Comput Biol. 2026 Jul 31;22(7):e1014570. doi: 10.1371/journal.pcbi.1014570 (PMC13450862; doi:10.1371/journal.pcbi.1014570)
Supplement: Supplementary file 3 [file pcbi.1014570.s003.pdf]

Reviewer Attachment

I thank the authors for addressing my previous comments. I have no further questions or concerns regarding the revised manuscript.
