# Supplementary material for: NLCD: A method to discover nonlinear causal relations among genes
Source: PLoS Comput Biol. 2026 Jul 31;22(7):e1014570. doi: 10.1371/journal.pcbi.1014570 (PMC13450862; doi:10.1371/journal.pcbi.1014570)
Supplement: Peer review history

# Peer review: Decision Letter 0

**DOI:** 10.1371/journal.pcbi.1014570.r001

**Authors:** Pedro Mendes, Yuehua Cui

25 Mar 2026

NLCD: A method to discover nonlinear causal relations among genes

PLOS Computational Biology

Dear Dr. Narayanan,

Thank you for submitting your manuscript to PLOS Computational Biology. After careful consideration, we feel that it has merit but does not fully meet PLOS Computational Biology's publication criteria as it currently stands. Therefore, we invite you to submit a revised version of the manuscript that addresses the points raised during the review process.

Please submit your revised manuscript by May 25 2026 11:59PM. If you will need more time than this to complete your revisions, please reply to this message or contact the journal office at ploscompbiol@plos.org. When you're ready to submit your revision, log on to https://www.editorialmanager.com/pcompbiol/ and select the 'Submissions Needing Revision' folder to locate your manuscript file.

Please include the following items when submitting your revised manuscript:

* A letter that responds to each point raised by the editor and reviewer(s). You should upload this letter as a separate file labeled 'Response to Reviewers'. This file does not need to include responses to formatting updates and technical items listed in the 'Journal Requirements' section below.

* A marked-up copy of your manuscript that highlights changes made to the original version. You should upload this as a separate file labeled 'Revised Manuscript with Track Changes'.

* An unmarked version of your revised paper without tracked changes. You should upload this as a separate file labeled 'Manuscript'.

If you would like to make changes to your financial disclosure, competing interests statement, or data availability statement, please make these updates within the submission form at the time of resubmission. Guidelines for resubmitting your figure files are available below the reviewer comments at the end of this letter

We look forward to receiving your revised manuscript.

Kind regards,

Yuehua Cui, PhD

Academic Editor

PLOS Computational Biology

Pedro Mendes

Section Editor

PLOS Computational Biology

**Journal Requirements:**

1) Please ensure that the CRediT author contributions listed for every co-author are completed accurately and in full.

At this stage, the following Authors/Authors require contributions: Aravind Easwar, and Manikandan Narayanan. Please ensure that the full contributions of each author are acknowledged in the "Add/Edit/Remove Authors" section of our submission form.

The list of CRediT author contributions may be found here: https://journals.plos.org/ploscompbiol/s/authorship#loc-author-contributions

2) We ask that a manuscript source file is provided at Revision. Please upload your manuscript file as a .doc, .docx, .rtf or .tex. If you are providing a .tex file, please upload it under the item type u2018LaTeX Source Fileu2019 and leave your .pdf version as the item type u2018Manuscriptu2019.

3) Please provide an Author Summary. This should appear in your manuscript between the Abstract (if applicable) and the Introduction, and should be 150-200 words long. The aim should be to make your findings accessible to a wide audience that includes both scientists and non-scientists. Sample summaries can be found on our website under Submission Guidelines:

https://journals.plos.org/ploscompbiol/s/submission-guidelines#loc-parts-of-a-submission

4) Please upload all main figures as separate Figure files in .tif or .eps format. For more information about how to convert and format your figure files please see our guidelines:

https://journals.plos.org/ploscompbiol/s/figures

5) We have noticed that you have uploaded Supporting Information files, but you have not included a list of legends. Please add a full list of legends for your Supporting Information files after the references list.

6) Please amend your detailed Financial Disclosure statement. This is published with the article. It must therefore be completed in full sentences and contain the exact wording you wish to be published.

State what role the funders took in the study. If the funders had no role in your study, please state: "The funders had no role in study design, data collection and analysis, decision to publish, or preparation of the manuscript.".

If you did not receive any funding for this study, please simply state: u201cThe authors received no specific funding for this work.u201d

**Reviewers' comments:**

Reviewer's Responses to Questions

**Comments to the Authors:**

**Please note here if the review is uploaded as an attachment.**

Reviewer #1: I have a few comments and suggestions that I think would help clarify the contribution and improve the overall presentation of the manuscript:

1. The idea of extending CIT to handle nonlinear relationships is very interesting and well motivated. It would help to more clearly explain how NLCD differs from other nonlinear or ML-based causal discovery methods, and what it adds beyond existing approaches.

2. The use of KRR, SVR, and neural networks is reasonable, and KRR appears to perform best in the benchmarks. Please explain why KRR was chosen as the default, and how sensitive results are to tuning or kernel choice.

3. For me it would be helpful to include a clearer check of p-value calibration or type I error control, particularly for nonlinear but non-causal settings.

4. Conditional feature importance is a key part of the method. A bit more intuition about when CFI works well, and when it might struggle (for example, when there is little overlap between genotype groups), would improve clarity. Also since the proposed method involves nonlinear regression and many permutations, some discussion of computation time can help readers understand when NLCD is practical to use.

5. The GTEx application is interesting, but some assessment of robustness (e.g., sensitivity to permutations, model choice) would increase confidence in these findings. In addition, a bit more discussion of biological plausibility would strengthen the real-data section.

Reviewer #2: The authors propose a method called NLCD, which extends the classical CIT framework to the nonlinear setting, and demonstrate its application to both human and yeast data. The manuscript is well written, and most methodological components are clearly explained. I have several questions and suggestions that may help further clarify and strengthen the work.

1. How does NLCD perform under a structure such as B←L→A, and at the same time A->B? In an MR framework, this corresponds to an invalid IV scenario where the instrument directly affects the outcome conditional on the exposure (horizontal pleiotropy).

2. Since NLCD is built upon the CIT framework proposed by Millstein et al. (2009), it is important to clearly explain the rationale for the four tests described in Section 2.2.4. In particular, the exact null and alternative hypotheses for each test should be explicitly stated. This would make the logical structure of the procedure more transparent and help readers understand how the joint decision rule corresponds to the overall causal hypothesis.

3. Regarding Test 1, why is a nonlinear model necessary in that step? For example, in eQTL analysis, a simple linear regression is often sufficient to test the association between a variant and expression.

4. Another concern relates to potential overfitting. If the same dataset is used for both training the nonlinear model and evaluating the test statistic, this may introduce optimism bias. Have the authors considered cross-validation or an independent test set to construct the test statistic?

5. In the yeast data application, the results suggest that NLCD does not outperform the original CIT, and even in the subset results, it is comparable to the CIT. Does this indicate that NLCD did not perform well in detecting linear relationship? It would be informative to compare NLCD and CIT at the level of the four component tests, for example by identifying which specific tests fail or differ between the two methods.

6. For human data application, how do other SOTA methods perform in this application?

7. In Figure 5b–c, the authors state that “the NLCD predictions for all three values of L are close to each other in the overlap region,” whereas in Figure 5d–e they indicate that predictions differ across values of L in the overlap region. However, the y-axis scales are not the same across panels, which makes it difficult to visually assess these differences. Clarification would be helpful. In addition, please specify what the colors of the dots represent in these plots.

8. Since the real-data results are largely comparable to existing methods, it would be helpful to briefly clarify in what settings NLCD is expected to provide clear advantages, for example, under stronger nonlinear relationships or more complex traits, and whether its intended scope extends beyond gene-level applications.

Reviewer #3: This work represents a non-trivial development of a CIT-type mediation analysis approach for non-linear settings, particularly involving gene regulatory networks. The approach is well thought-out, implemented, and the rationale is clearly explained in the manuscript. Simulations demonstrate that the approach (NLCD) out performs the CIT, Findr, and MRPC in simulated non-linear scenarios and performs competitively to the CIT in linear settings. Software is provided via GitHub. It's my opinion that this will be an important and useful tool for the scientific community for identifying causal relationships between molecular features in non-linear settings, previously a methodological gap in this field.

The manuscript is well written and organized.

I have one comment that the authors could address, and that involves heterogeneity or experimental design-related factors that might need to be considered during the analysis. For example, what if there are batches, multiple tissues, cell types, genders, etc. How can this approach accommodate adjustment covariates?

Minor comment: Change section 2.2 title from “Our NLCD Method” to something more informative, e.g., “NLCD Algorithm”

**********

**Have the authors made all data and (if applicable) computational code underlying the findings in their manuscript fully available?**

The PLOS Data policy  requires authors to make all data and code underlying the findings described in their manuscript fully available without restriction, with rare exception (please refer to the Data Availability Statement in the manuscript PDF file). The data and code should be provided as part of the manuscript or its supporting information, or deposited to a public repository. For example, in addition to summary statistics, the data points behind means, medians and variance measures should be available. If there are restrictions on publicly sharing data or code —e.g. participant privacy or use of data from a third party—those must be specified.

Reviewer #1: Yes

Reviewer #2: None

Reviewer #3: Yes

**********

PLOS authors have the option to publish the peer review history of their article (what does this mean? ). If published, this will include your full peer review and any attached files.

If you choose “no”, your identity will remain anonymous but your review may still be made public.

**Do you want your identity to be public for this peer review?**   For information about this choice, including consent withdrawal, please see our Privacy Policy .

Reviewer #1: No

Reviewer #2: No

Reviewer #3: **Yes:**   Joshua Millstein

[NOTE: If reviewer comments were submitted as an attachment file, they will be attached to this email and accessible via the submission site. Please log into your account, locate the manuscript record, and check for the action link "View Attachments". If this link does not appear, there are no attachment files.]

**Figure resubmission:**

**Reproducibility:**

?>

# Author comment: Author response to Decision Letter 1

**DOI:** 10.1371/journal.pcbi.1014570.r002

18 Jun 2026

Attachment Submitted filename: NLCD_point_by_point_responses.pdf

# Peer review: Decision Letter 1

**DOI:** 10.1371/journal.pcbi.1014570.r003

**Authors:** Pedro Mendes, Yuehua Cui

13 Jul 2026

Dear Dr. Narayanan,

We are pleased to inform you that your manuscript 'NLCD: A method to discover nonlinear causal relations among genes' has been provisionally accepted for publication in PLOS Computational Biology.

Before your manuscript can be formally accepted you will need to complete some formatting changes, which you will receive in a follow up email. A member of our team will be in touch with a set of requests.

Please note that your manuscript will not be scheduled for publication until you have made the required changes, so a swift response is appreciated.

IMPORTANT: The editorial review process is now complete. PLOS will only permit corrections to spelling, formatting or significant scientific errors from this point onwards. Requests for major changes, or any which affect the scientific understanding of your work, will cause delays to the publication date of your manuscript.

Should you, your institution's press office or the journal office choose to press release your paper, you will automatically be opted out of early publication. We ask that you notify us now if you or your institution is planning to press release the article. All press must be co-ordinated with PLOS.

Thank you again for supporting Open Access publishing; we are looking forward to publishing your work in PLOS Computational Biology.

Best regards,

Yuehua Cui, PhD

Academic Editor

PLOS Computational Biology

Pedro Mendes

Section Editor

PLOS Computational Biology

***********************************************************

Reviewer's Responses to Questions

**Comments to the Authors:**

**Please note here if the review is uploaded as an attachment.**

Reviewer #1: The authors have adequately addressed my previous comments, and I have no further questions or concerns.

Reviewer #2: The authors have addressed all my comments and I have no further comments.

Reviewer #3: The authors have adequately addressed my concerns.

**********

**Have the authors made all data and (if applicable) computational code underlying the findings in their manuscript fully available?**

The PLOS Data policy  requires authors to make all data and code underlying the findings described in their manuscript fully available without restriction, with rare exception (please refer to the Data Availability Statement in the manuscript PDF file). The data and code should be provided as part of the manuscript or its supporting information, or deposited to a public repository. For example, in addition to summary statistics, the data points behind means, medians and variance measures should be available. If there are restrictions on publicly sharing data or code —e.g. participant privacy or use of data from a third party—those must be specified.

Reviewer #1: None

Reviewer #2: None

Reviewer #3: None

**********

PLOS authors have the option to publish the peer review history of their article (what does this mean? ). If published, this will include your full peer review and any attached files.

If you choose “no”, your identity will remain anonymous but your review may still be made public.

**Do you want your identity to be public for this peer review?**   For information about this choice, including consent withdrawal, please see our Privacy Policy .

Reviewer #1: No

Reviewer #2: No

Reviewer #3: **Yes:**   Joshua Millstein

Attachment Submitted filename: reviewer_attachment_response.pdf

# Editor report: Acceptance letter

**DOI:** 10.1371/journal.pcbi.1014570.r004

**Authors:** Pedro Mendes, Yuehua Cui

PCOMPBIOL-D-26-00257R1

NLCD: A method to discover nonlinear causal relations among genes

Dear Dr Narayanan,

I am pleased to inform you that your manuscript has been formally accepted for publication in PLOS Computational Biology. Your manuscript is now with our production department and you will be notified of the publication date in due course.

The corresponding author will soon be receiving a typeset proof for review, to ensure errors have not been introduced during production. Please review the PDF proof of your manuscript carefully, as this is the last chance to correct any errors. Please note that major changes, or those which affect the scientific understanding of the work, will likely cause delays to the publication date of your manuscript.

Soon after your final files are uploaded, unless you have opted out, the early version of your manuscript will be published online. The date of the early version will be your article's publication date. The final article will be published to the same URL, and all versions of the paper will be accessible to readers.

For Research, Software, and Methods articles, you will receive an invoice from PLOS for your publication fee after your manuscript has reached the completed accept phase. If you receive an email requesting payment before acceptance or for any other service, this may be a phishing scheme. Learn how to identify phishing emails and protect your accounts at https://explore.plos.org/phishing.

Thank you again for supporting PLOS Computational Biology and open-access publishing. We are looking forward to publishing your work!

With kind regards,

Janani Seenivasan

PLOS Computational Biology | Carlyle House, Carlyle Road, Cambridge CB4 3DN | United Kingdom ploscompbiol@plos.org | Phone +44 (0) 1223-442824 | ploscompbiol.org | @PLOSCompBiol
